# Effects of Cholesterol on the Breast Cancer Resistance Protein: Studies through the Synthesis and Biological Evaluation of Chemical Tools

**DOI:** 10.1002/cmdc.202400712

**Published:** 2025-01-31

**Authors:** Ingrid Fatima Zattoni, Bruna Estelita Rugisnk, Isadora da Silva Zanzarini, Alan Guilherme Gonçalves, Vivian Rotuno Moure, Glaucio Valdameri, Ahcène Boumendjel

**Affiliations:** ^1^ Graduate Program in Pharmaceutical Sciences Laboratory of Cancer Drug Resistance Federal University of Parana Curitiba 80210-170, PR Brazil; ^2^ Life Sciences and Medical School Pontifical Catholic University of Paraná Curitiba Brazil; ^3^ Department of Pharmacy Federal University of Parana Curitiba, PR Brazil; ^4^ Univ. Grenoble Alpes INSERM LRB 38000 Grenoble France

**Keywords:** ABCG2, Chemoresistance, Cholesterol, Cholesterol conjugates, Chemical tools

## Abstract

The breast cancer resistance protein (BCRP/ABCG2) plays a major role in the multidrug resistance of cancers toward chemotherapeutic treatments. It was demonstrated that cholesterol regulates the ABCG2 activity, suggesting that lower levels of membrane cholesterol decrease the ABCG2 activity in mammalian cells. However, the precise mechanism remains unclear. To better understand the role of cholesterol in the ABCG2 activity, we studied the ABCG2‐mediated efflux of different substrates in the presence of different concentrations of cholesterol. Moreover, we synthetized derivatives of cholesterol linked either to known ABCG2 inhibitors or fluorescents probes. A chalcone‐cholesterol was synthetized to investigate the influence of cholesterol on ABCG2 inhibition, and a BODIPY‐cholesterol was developed to track cholesterol trafficking on mammalian cells and investigate the behavior of cholesterol as an ABCG2 substrate. The obtained results with three different substrates of ABCG2 showed that cholesterol did not affect the intracellular amount of substrates nor the transport activity.

## Graphical abstract

This study explores the role of cholesterol in the regulation of the efflux pump ABCG2. Substrates and inhibitors of ABCG2, cholesterol linked to fluorescents probes were synthesized and tested to investigate the influence of cholesterol on ABCG2 inhibition and to track cholesterol trafficking on cells. We show that cholesterol did not affect the intracellular amount nor the transport activity of ABCG2.

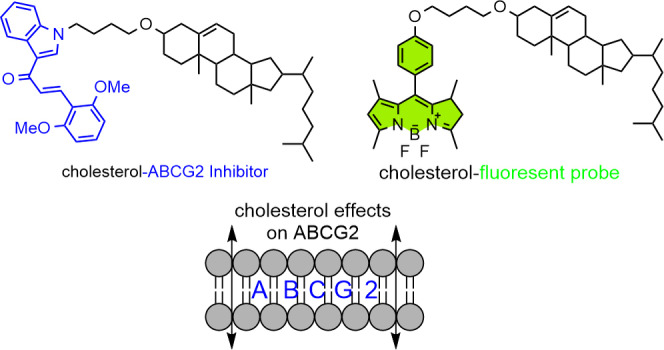

## Introduction

Overexpression of ABC transporters in cancer cells is described as a prominent cause of multidrug resistance (MDR).^[1]^ However, ABC transporters also have important physiological functions.^[2]^ The human genome encodes 48 ABC proteins, divided in seven subfamilies (A–G) based on the sequence of their nucleotide binding domain.^[3]^ Amongst their physiological roles are detoxification properties on liver, kidney, placenta[^4^, ^5^] and transport of endogenous ligands, such as, cholesterol and urate.[^6^, ^7^, ^8^] The ABC transporters ABCA1 and ABCG1/5/8 are widely known as cholesterol transporters.^[9]^ ABCA1 is related to regulation of cholesterol membrane content, delivering cholesterol from cell surface to HDL lipoprotein, participating on cholesterol reverse transport to liver.^[6]^ On the other hand, ABCG5/8 can be found on enterocytes, hepatocytes and gallbladder epithelium, removing cholesterol and other sterols from cytoplasm.^[7]^

The importance of cholesterol for ABCG2 activity is also described.^[10]^ There is evidence demonstrating the importance of cholesterol for ABCG2 functioning and the capacity of ABCG2 to efflux cholesterol‐related compounds.^[11]^ It was reported that ABCG2 can efflux cholesterol related compounds, like sulfated steroids estrone‐3‐sulfate (E_3_S) and dehydroepiandrosterone sulfate (DHEAS), despite the unknown physiological significance.^[12]^ Moreover, it was demonstrated that the depletion of cell membrane cholesterol decreased ABCG2 activity on mammalian cells and the loading of cholesterol in SF9 insect cells increased ATPase activity and estrone‐3‐sulfate efflux.[^10^, ^13^] Also, ABCG2 was found in lipid/cholesterol rich membrane regions, like lipid rafts, and the reduction of cholesterol content resulted in reduction of ABCG2 activity.^[14]^ Those studies suggest that cholesterol may be important for ABCG2 optimal activity by enabling dimerization, or influencing binding process of small substrates.[^14^, ^15^]

Since a variety of ABC transporters can interact with cholesterol, either as substrate or increasing the transport activity,^[16]^ it was proposed a “fill‐in” model, in which cholesterol would assist the efflux of small molecules by filling the binding sites empty spaces.[^17^, ^18^, ^19^] The importance of cholesterol efflux for physiological events was also demonstrated on spermatozoa maturation.^[20]^ The efflux of cholesterol in those cells is important to modulate membrane fluidity during capacitation and may be mediated by ABC transporters, like ABCG2, previously identified in spermatozoa acrosome.^[21]^ Since cholesterol is a non‐fluorescent compound, authors used BODIPY‐cholesterol as a way to track cholesterol modifications on spermatozoids, proposing this modified cholesterol as a reliable probe to track cholesterol trafficking on spermatozoids.^[22]^

Some amino acids are important for cholesterol binding on ABCG2, including Arg482.^[10]^ Mutants on this position (Gly or Thr) showed that cholesterol had low effect on ABCG2 on human cells, but was fully active on SF9 membranes.[^23^, ^24^] ABCG2 mutants with hydrophobic or positively charged amino acids were highly dependent of cholesterol to optimal activity,^[25]^ however, mutants with polar, uncharged, or negatively charged amino acids suffered lower interference by cholesterol.^[26]^ Actually, in the last case, cholesterol did not increase the activity and the protein activity was practically unchanged.^[25]^ The ABCG2 structure obtained by Cryo‐electron microscopy enhanced our comprehension about ABCG2 and cholesterol interaction. Cholesterol was found to interact with TM2 (Phe432 and Phe439) and TM5a (Leu539, Leu543, Val546 and Met549).^[27]^ Those amino acids residues are related with cavity 1, which is the binding region of different substrates and inhibitors.[^28^, ^29^] Together, those studies suggests that cholesterol and its derivatives modulate ABCG2 activity[^10^, ^13^, ^25^] and propose that steroids compounds, such as bile acids, may be transported by ABCG2.^[25]^ In summary, there is accumulated evidences indicating interactions between ABCG2 and cholesterol. However, data is needed to better validate or invalidate and understand such interactions.

**Figure 1:**
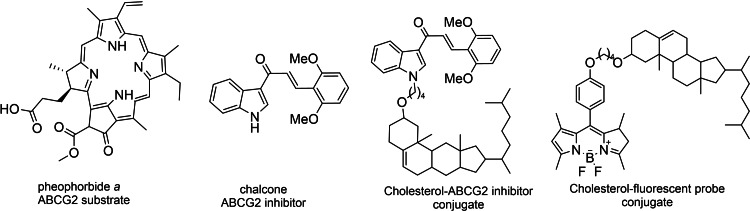
Structures of chemical tools synthesized and used in the study.

In order to evaluate the interference of cholesterol on ABCG2 activity, we conducted full study with substrates of ABCG2 in absence or presence of cholesterol, cholesterol in presence or absence of ABCG2 inhibitors, cholesterol linked to a fluorescent probe to track its trafficking. Herewith, we synthetized four chemical tools: pheophorbide *a*, an ABCG2 substrate; a chalcone as ABCG2 inhibitor, cholesterol linked to a known ABCG2 inhibitor; cholesterol linked to a fluorescent probe to track cholesterol trafficking on mammalian cells (Figure 1).

## Results and Discussion

### Chemistry

**Scheme 1:**
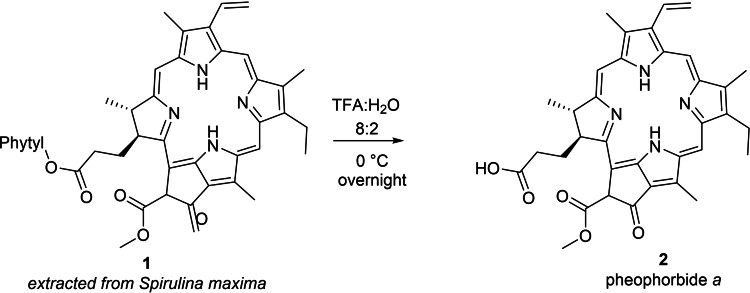
Preparation of pheophorbide *a*.

Pheophorbide *a* was prepared in one step by the hydrolysis of Pheophorbide phytyl ester which was extracted from *Spirulina maxima* (Scheme 1).^[30]^

**Scheme 2:**
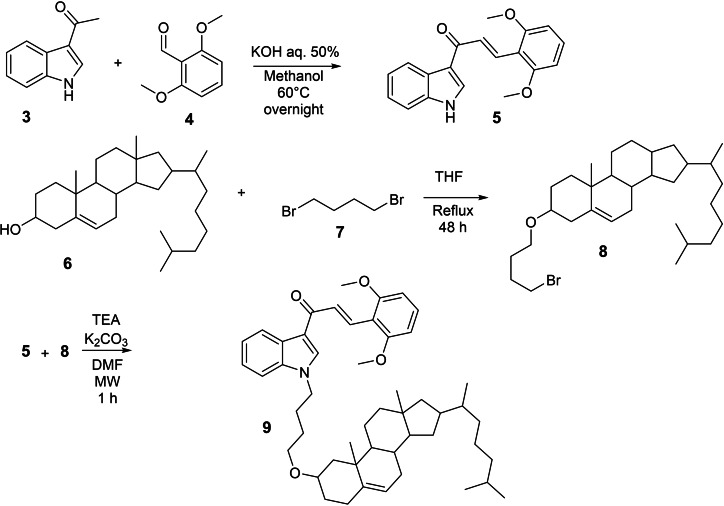
Synthesis of chalcone‐cholesterol conjugate.

Chalcone **5** was selected because it was previously identified as a potent inhibitor of ABCG2.^[31]^ Chalcone **5** was prepared in one step from the commercially available acetyl indole (**3**) and 2,6‐dimethoxybenzaldehyde (**4**) in the presence of potassium hydroxide, with 66 % yield (Scheme 2).

The synthesis of the inhibitor‐cholesterol conjugate was accomplished according to Scheme 2. The linear C‐4 chain was chosen as a linker since it was previously found as the optimal length in the preparation of chalcone‐derived dimers as ABCG2 inhibitors. Cholesterol was reacted with 1,4‐dibromobutane to afford the monoalkylated derivative **8** (16 % yield).^[32]^ The final step was the reaction of **5** with **8** in the presence of a mixture of trimethylamine and potassium carbonate to provide the final conjugate **9** in 7 % yield.

**Scheme 3:**
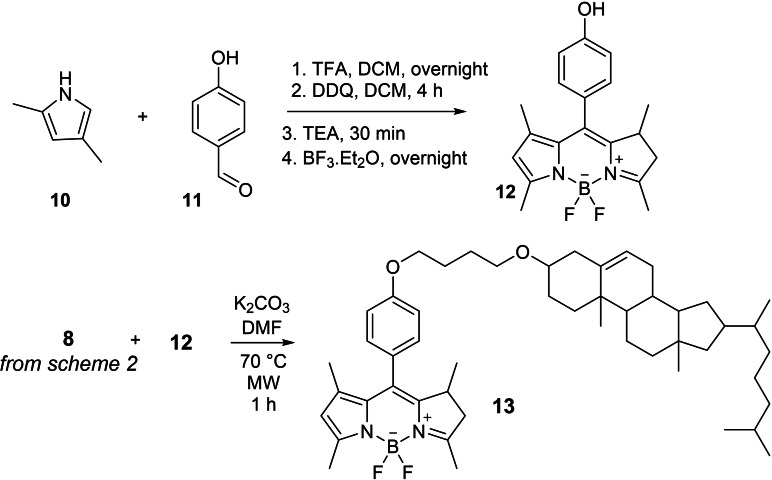
Synthesis of BODIPY‐cholesterol conjugate.

The BODIPY‐cholesterol conjugate was obtained as shown in Scheme 3. BODIPY (**12**) was prepared from 2,4‐dimethylpyrole **10** and 4‐hydroxybenzaldehyde **11**, following a four reaction sequence, involving: a condensation reaction catalyzed by TFA, an oxidation with DDQ, and subsequent treatment with base and BF_3_.Et_2_O.^[33]^ BODIPY **12** was reacted with alkylated cholesterol **8**, under basic conditions, to afford the expected compound **13** in 7 % yield.

### Influence of Cholesterol on ABCG2‐mediated Drug Efflux

**Figure 2:**
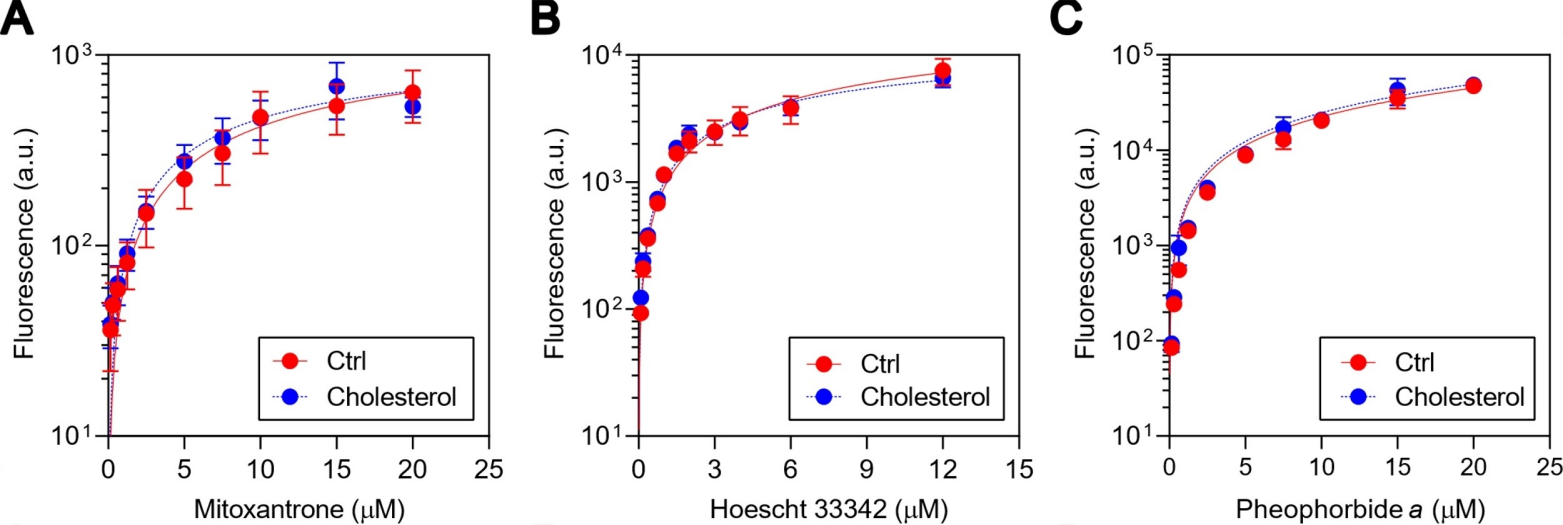
Influence of cholesterol on ABCG2‐mediated transport. ABCG2 efflux behavior in the presence and absence of 50 μM of cholesterol and substrates mitoxantrone (0.15–20 μM), hoechst 33342 (0.09–12 μM) and pheophorbide *a* (0.15–20 μM) for 30 minutes. Experiment performed by flow cytometry using HEK293‐*ABCG2* cell line. Data represent the mean ± SD of at least three replicates.

Cholesterol seems to regulate ABCG2 activity.^[25]^ It was suggested that lower levels of membrane cholesterol decrease the ABCG2 activity in mammalian cells,^[10]^ and the loading of cholesterol in SF9 insect cells increases its activity.^[13]^ However, the precise mechanism remains unclear. To verify the role of cholesterol in the ABCG2 activity, the ABCG2‐mediated efflux of different substrates (mitoxantrone, hoechst 33342 and pheophorbide *a*) was measured in presence or absence of a saturating concentration of cholesterol (50 μM). Stably transfected cells overexpressing ABCG2 (HEK293‐*ABCG2*) were exposed to increasing concentrations of the commercial ABCG2 substrates mitoxantrone and hoechst 33342. As shown in Figure 2A, the presence of cholesterol did not affect the intracellular amount of mitoxantrone. The same pattern was observed for the substrate hoechst 33342 (Figure 2B). To confirm this effect, we extracted pheophytin from *Spirulina maxima* to subsequently produce pure pheophorbide *a* (see Experimental Section), a specific non‐commercial ABCG2 substrate. The absence of effect of cholesterol on ABCG2‐mediated pheophorbide *a* efflux (Figure 2C) supports the findings observed with the other substrates, that at least for human cells, the loading of additional cholesterol did not affect the ABCG2 activity, suggesting that the amount of cholesterol into membrane is optimal for the transport activity.

### Influence of Cholesterol on ABCG2 Inhibition

Considering the data that external cholesterol did not affect ABCG2, but structural studies revealed the presence of cholesterol in the transmembrane region responsible for the binding of substrates and inhibitors of ABC transporters,[^28^, ^29^] we hypothesized that conjugates of well‐known ABCG2 ligands and cholesterol could improve the activity of ABCG2 inhibitors.

**Figure 3:**
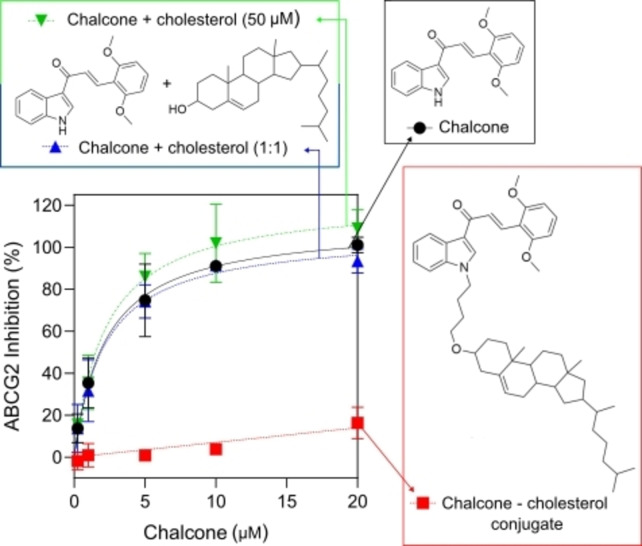
Influence of free Cholesterol (**6**) and covalently bounded conjugate (**9**) on inhibition. **3A**. Structure of compounds: Chalcone (**5**), Cholesterol (**6**) and conjugate (**9**). **3B**. Experiment was performed by flow cytometry (UV 450–50 filter) using HEK293‐*ABCG2* cell line, Ko143 (0.5 μM) as inhibition control hoechst 33342 (3 μM) as substrate. Data reflect three independent experiments.

To investigate this hypothesis, a new chalcone derivative was synthesized based on previous findings.^[34]^ As shown in Figure 3, this chalcone inhibited ABCG2, showing an EC_50_ value (concentration that cause a half of maximal inhibition) of 2.21 μM. To verify the external loading of cholesterol, an EC_50_ curve of inhibition was performed in presence of cholesterol (50 μM). The presence of external cholesterol did not affect the potency of inhibition, since the EC_50_ value in presence of cholesterol was 2.14 μM (Table 1). In addition, a curve using increasing concentrations of chalcone and external cholesterol at ratio 1 : 1 from 0.25–20 μM also did not affect the inhibition effect (Figure 3 and Table 1). The EC_50_ curves also revealed that this chalcone derivative causes a complete ABCG2 inhibition, since the I_MAX_ values (maximal values of ABCG2 inhibition) were around 100 % of inhibition, as compared to the reference inhibition Ko143 (Table 1).

**Table 1:** EC_50_ and I_MAX_ values for ABCG2 inhibition.

Compounds tested	IC_50_ (μM) ± SD	I_MAX_ (%) ± SD
Chalcone (**5**)	2.21±0.50	111±6.29
Chalcone (**5**) 0.25–20 μM + Cholesterol (**6**) 50 μM	2.14±0.49	106±6.03
Chalcone (**5**) 0.25–20 μM + Cholesterol (**6**) 0.25–20 μM	2.21±0.55	123±7.60
Conjugate (**9**) 0.25–20 μM	No inhibition	No inhibition

To evaluate the conjugate strategy, chalcone (**5**) was covalently bound to cholesterol (6). Interestingly, the chalcone‐cholesterol conjugate completely abrogated the inhibitory effect of the chalcone (Figure 3B and Table 1). Despite some promising results of the use of conjugates targeting ABCG2,[^35^, ^36^] the use of cholesterol is prejudicial. We hypothesized that cholesterol may either ubiquitously anchor chalcone into cell membranes, preventing the intracellular internalization of the inhibitor and consequently its interaction in the ABCG2 binding site, or cholesterol exerts steric hindrance on ABCG2, also preventing the chalcone interaction.

### Cellular Localization of Cholesterol Conjugate

**Figure 4:**
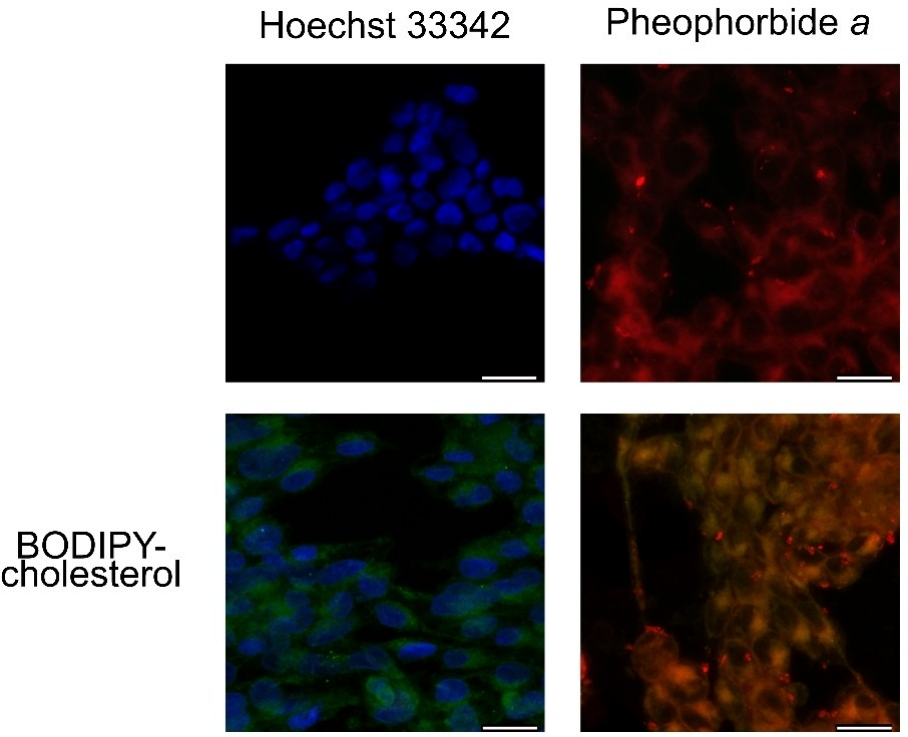
Cell accumulation of BODIPY‐cholesterol in HEK293‐*ABCG2* by confocal microscopy. Cells were exposed to Hoechst 33342 (3 μM) to nuclear stain, and pheophorbide *a* (20 μM) to cytoplasm stain (in presence of Ko143 (1 μM) for ABCG2 inhibition). Co‐treatment of these dyes with BODIPY‐cholesterol (20 μM) was used to assess cell localization of the conjugate. The scale bar indicates 20 μm.

Since the chalcone‐cholesterol conjugate cannot be easily tracked, we synthesized a fluorescent dye (BODIPY) to produce a fluorescent conjugate of BODIPY‐cholesterol. To investigate the cellular accumulation of BODIPY‐cholesterol by confocal microscopy, additional staining with hoechst 33342 and pheophorbide *a* were used.^[37]^ Since HEK293‐*ABCG2* cells were used, inhibition controls using Ko143 were also performed. As shown in Figure 4, the nucleus was stained by hoechst (blue fluorescence) and the mitochondria/cytoplasm was stained by pheophorbide *a* (red fluorescence). The merge images between BODIPY‐cholesterol either with hoechst or with pheophorbide *a* showed that 30 minutes are enough for the conjugate internalization (Figure 4). ABCG2 inhibition by Ko143 caused an intracellular accumulation of hoechst and pheophorbide *a* (Figure S1). In addition, it was observed a subcellular localization of the conjugate in the cytoplasm (Figures 4 and Figure S1), suggesting that cholesterol conjugates can cross the cell membrane but drag the inhibitors outside of the ABCG2 drug binding site.

### BODIPY‐Cholesterol Transport by ABCG2

To investigate a possible ABCG2‐mediated transport of cholesterol, the fluorescent conjugate of BODIPY‐cholesterol was used. Free BODIPY showed a very high fluorescence at 4 μM; however, BODIPY‐cholesterol fluorescence was less intense (Figure S2). The optimized concentration for further studies by flow cytometry was 8 μM, based on the lowest concentration capable to separate the control condition (non‐fluorescent cells) from BODIPY‐cholesterol treated cells (Figure S2). Considering that BODIPY‐cholesterol can trespass cellular membrane, it was firstly investigated if free cholesterol could affect the cellular uptake of BODIPY‐cholesterol. As shown in Figure S3, the cellular uptake of BODIPY‐cholesterol was not affected even at ratio 10‐fold higher of free cholesterol.

**Figure 5:**
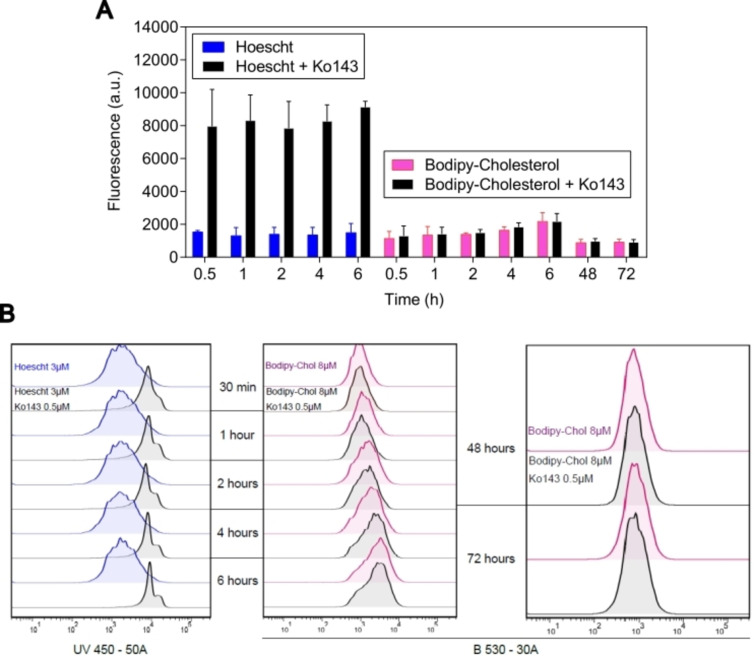
(**A**) Intracellular fluorescence of hoechst 33342 (3 μM) and BODIPY‐cholesterol conjugate (8 μM) in the presence and absence of Ko143 (1 μM). (**B**) Histograms of hoechst 33342 and Ko143; BODIPY‐cholesterol conjugate and Ko143; BODIPY‐cholesterol conjugate and Ko143 after 48 and 72 hours.

Finally, to verify a possible transport of BODIPY‐cholesterol mediated by ABCG2, HEK293‐*ABCG2* cells were treated for 30 minutes with either the substrate hoechst or the fluorescent conjugate in two conditions: absence and presence of the reference ABCG2 inhibitor Ko143. After, the monolayer of cells was washed with PBS and the medium was replaced. The flow cytometry analysis was performed at different periods of times (30 minutes, 1, 2, 4 and 6 hours after washing). As expected, hoechst 33342 fluorescence was higher in the presence of inhibitor, confirming the substrate behavior (Figure 5A). In the same conditions, BODIPY‐cholesterol fluorescence was equivalent in the presence or absence of Ko143, suggesting that this conjugate is not transported by ABCG2 (Figure 5). An efflux mediated by ABCG2 was also investigated by confocal microscopy and confirmed that the presence of Ko143 did not affect the cellular accumulation of the BODIPY‐cholesterol conjugate (Figure S1). Further, a long‐term experiment was performed, allowing a possible transport mediated by ABCG2 for 48 and 72 hours. Even in this condition, the intracellular fluorescence of BODIPY‐cholesterol was equivalent in the presence or absence of Ko143, confirming that BODIPY‐cholesterol is not an ABCG2 substrate (Figure 5A and B).

## Conclusions

The relevance of cholesterol for ABCG2 activity is undoubtedly important. This study focused on finding evidence concerning the use of cholesterol to produce conjugates with ABCG2 inhibitors, and to evaluate a possible efflux of cholesterol mediated by ABCG2. Thus, two conjugates were synthetized for the first time: a chalcone‐cholesterol (targeting ABCG2 inhibition), and a BODIPY‐cholesterol (to be used as a fluorescent probe). Interestingly, the chalcone‐cholesterol conjugate failed to inhibit ABCG2, fully abrogating the inhibition effect of the chalcone alone. In addition, the BODIPY‐cholesterol conjugate was not transported by ABCG2.

Further investigations are essential to understand the cholesterol's influence on ABCG2 activity. One promising direction involves the design and synthesis of new cholesterol conjugates, employing alternative linkers or chemical modifications to overcome steric limitations and improve cellular uptake. Additionally, molecular dynamics simulations could provide insights into the interaction between cholesterol or its derivatives and ABCG2, helping to clarify the binding mechanisms and the specific amino acid residues involved in cholesterol recognition.

Complementary approaches, such as lipidomics and membrane composition analyses, could further elucidate how variations in membrane lipid content affect ABCG2 activity. Moreover, fluorescent probes like BODIPY‐cholesterol should be applied to advanced models, such as 3D cell cultures or in vivo systems, to capture more detailed and physiologically relevant cholesterol dynamics. Addressing these experimental gaps has the potential to yield profound insights into the mechanisms underlying cholesterol's regulation of ABCG2 activity.

## Experimental Section

**Chemistry**: All reagents and solvents were reagent grade and used without previous purification. The chromatography was performed using a pre‐coated TLC sheets ALUGRAM Xtra SIL G/UV 254 with 0.2 mm silica gel 60 layer with fluorescent indicator UV 254. The elution was performed using the mixture of solvents indicated for each reaction and revealed with UV light or TLC revealing solution as indicated. The ^1^H and ^13^C NMR were acquired using a Bruker Avance 400 MHz and Bruker Avance III 500 MHz spectrometers. The samples were dissolved on the adequate deuterium solvent and the respective solvent signal was used as internal standard. The spectra interpretation was performed using MestReNova software (version 6.0.2‐5475, Mastrelab Research S.L). The mass spectrometry was performed on a BRUKER amaZon speed and Thermo Scientific LTQ Orbitrap XL for high resolution spectra, both with electrospray as ionization source. A CEM Discovery microwave was used for microwave assisted synthesis.

### Extraction of Pheophytin (1)

25 g of dried *Spirulina maxima* were mixed with 250 mL of acetone and stirred under reflux at 60 °C for three hours. Then, the extract was filtered to separate the organic phase and the powder was extracted two more times with the same amount of acetone. After, the extracts were combined and evaporated under reduced pressure, resulting in 1.7 g of dried extract. A chromatographic column was carried out using dichloromethane as eluent to remove the carotenoids (orange fraction) and dichloromethane: acetone (95 : 5) to elute the pheophytin (1). The product was isolated as a dark green solid (21 % of yield). Monoisotopic mass: calculated [M+H]^+^ 871.57320, found 871.57185, ^1^H NMR (Figure S4).

^1^H NMR (400 MHz, CDCl_3_) δ 9.48 (s, 1H), 9.33 (s, 1H), 8.55 (s, 1H), 7.96 (dd, *J*=17.8, 11.5 Hz, 1H), 6.31–6.23 (m, 2H), 6.19–6.14 (m, 1H), 5.41–5.32 (m, 2H), 5.22–5.08 (m, *J*=8.3, 7.1 Hz, 1H), 4.54–4.40 (m, *J*=19.5, 12.5, 7.0 Hz, 3H), 4.25–4.18 (m, 1H), 3.90 (s, 3H), 3.69–3.60 (m, 5H), 3.39 (s, 3H), 3.19 (s, 3H), 2.41–2.30 (m, 2H), 2.26–2.14 (m, *J*=20.7, 10.3, 5.3 Hz, 2H), 1.92–1.85 (m, *J*=12.1, 7.0 Hz, 2H), 1.82 (d, *J*=7.3 Hz, 3H), 1.68 (t, *J*=6.3 Hz, 3H), 1.58 (s, 3H), 0.85 (d, *J*=6.6 Hz, 6H), 0.83–0.77 (m, 6H), −1.64 (s, 1H).

### Synthesis of Pheophorbide *a* (2)

100 mg of purified Pheophytin (**1**) were mixed with 10 mL TFA: H_2_O (8 : 2 previously degassed with argon for 30 minutes). The reaction was stirred overnight in ice bath and protected from light. Then, 60 mL of distilled water and 60 mL of dichloromethane were added to the reaction to extract the product. The organic layer was separated and washed 3 times with water and one time with a NaHCO_3_ 10 % (60 mL each time, a precipitate is formed in this step). Then, 60 mL of citric acid 10 % was added carefully to wash the organic layer until the precipitate disappearance. Then, the dichloromethane was dried over anhydrous MgSO_4_ and evaporated under reduced pressure. The chromatographic column was performed using dichloromethane: ethyl acetate (1 : 1) as eluent to remove the starting material, then, dichloromethane: methanol (95 : 5) was used to elute the product. The product (**2**) was collected as a black solid (75 % yield). Monoisotopic mass: calculated [M+H]^+^ 593.2785, found 593.2759. ^1^H NMR (Figure S5).

^1^H NMR (400 MHz, CDCl_3_) δ 9.35 (s, 1H), 9.20 (s, 1H), 8.45 (s, 1H), 7.89–7.74 (m, 1H), 6.20–5.99 (m, 3H), 4.35 (dd, *J*=7.3, 1.8 Hz, 1H), 4.11 (d, *J*=8.7 Hz, 1H), 3.77 (s, 3H), 3.58–3.44 (m, 5H), 3.27 (s, 3H), 3.07 (s, 3H), 2.60–2.40 (m, 2H), 2.25–2.11 (m, 2H), 1.72 (d, *J*=7.3 Hz, 3H), 1.56 (t, *J*=7.6 Hz, 3H), −1.77 (s, 1H).

### Chalcone (5)

321.9 mg (2 mmol) of 3 acetyl indole (**3**) and 334.0 mg (2 mmol) of 2.6 dimethoxy benzaldehyde (**4**) were dissolved in 20 mL of methanol, then, 2 mL of KOH 50 % aqueous were added to the mixture. The reaction was stirred overnight at 60 °C. The solvent was evaporated under reduced pressure and ethyl acetate was added to allow precipitation of chalcone (**5**) which was obtained with 66 % of yield. Monoisotopic mass: calculated [M+H]^+^ 308.12812, found 308.12771. ^1^H and ^13^C NMR (Figure S6).

^1^H NMR (400 MHz, DMSO) δ 8.42 (s, 1H), 8.39–8.34 (m, 1H), 8.07 (d, *J*=15.9 Hz, 1H), 7.95 (d, *J*=15.9 Hz, 1H), 7.59–7.54 (m, 1H), 7.38 (t, *J*=8.4 Hz, 1H), 7.29–7.20 (m, 2H), 6.78 (d, *J*=8.4 Hz, 2H), 3.97 (s, 6H). ^13^C NMR (101 MHz, DMSO) δ 184.84, 173.89, 159.62, 137.20, 133.87, 131.17, 130.46, 126.49, 126.06, 122.77, 121.67, 121.55, 117.68, 112.40, 111.95, 104.13, 55.96, 25.63.

### *O*‐Alkylated Cholesterol (8)

Cholesterol (**6**) (1.93 g, 5 mmol), NaH (800 mg, 20 mmol) and 1,4 dibromobutane (**7**) (2.4 mL, 20 mmol) were dissolved in 25 mL of dried THF. The reaction was stirred under reflux for 2 days. After this, distilled water was carefully added to the reaction (around 25 mL), following an extraction with ethyl acetate (3×25 mL). The ethyl acetate was collected, dried over MgSO_4_ and removed under reduced pressure. The purification was performed using cyclohexane: ethyl acetate 1 : 1 as eluent to separate the product from the starting material. A second column was performed using cyclohexane: dichloromethane 99 : 1 to remove the 1,4‐dibromobutane. The final product was removed from silica using ethyl acetate. The product visualization was performed using solution of H_2_SO_4_ 10 % in methanol. The product was isolated with 16 % yield. ^1^H and ^13^C NMR (Figure S7).

^1^H NMR (400 MHz, CDCl_3_) δ 5.34 (d, *J*=5.3 Hz, 1H), 3.48 (t, *J*=5.9 Hz, 2H), 3.44 (t, *J*=6.8 Hz, 2H), 3.17–3.07 (m, 1H), 2.34 (ddd, *J*=13.2, 4.7, 2.2 Hz, 1H), 2.23–2.11 (m, 1H), 2.06–1.76 (m, 8H), 1.74–1.00 (m, 24H), 0.99 (s, 4H), 0.91 (d, *J*=6.6 Hz, 4H), 0.86 (dd, *J*=6.6, 1.7 Hz, 6H), 0.67 (s, 3H).^13^C NMR (101 MHz, CDCl_3_) δ 141.02, 121.57, 79.11, 66.91, 56.82, 56.21, 50.25, 42.36, 39.83, 39.56, 39.20, 37.30, 36.93, 36.23, 35.82, 33.81, 31.99, 31.93, 29.84, 28.83, 28.49, 28.27, 28.04, 24.33, 23.87, 22.85, 22.59, 21.11, 19.41, 18.76, 11.89.

### Chalcone‐Cholesterol Conjugate (9)

17.8 mg of chalcone (**5**), 27 mg of cholesterol bromide (**8**), 16.2 mg of K_2_CO_3_ and 50 μL of triethylamine were dissolved in 2 mL of dried DMF and poured in a microwave flask. The flask was placed on a microwave platform and allowed to react during 1 hour at 70 °C. Then, the solvent was removed under reduced pressure and the crude was purified using dichloromethane/acetone 98 : 2 as eluent. When necessary, another purification was performed using cyclohexane: ethyl acetate 3 : 1 to provide the desired compound with 7 % yield. Monoisotopic mass: calculated [M+H]^+^ 748.52994, found 748.52881. ^1^H and ^13^C NMR (Figure S8).

^1^H NMR (400 MHz, CDCl_3_) δ 8.46 (dd, *J*=6.1, 2.7 Hz, 0.67H), 8.37 (dd, *J*=6.2, 2.9 Hz, 0.41H), 8.17 (d, *J*=16.0 Hz, 0.62H), 7.80 (s, 0.5H), 7.78 (d, *J*=16.0 Hz, 1.19H), 7.42 (s, 0.32H), 7.28 (ddd, *J*=32.3, 6.1, 2.9 Hz, 2.58H), 7.18–7.13 (m, 0.88H), 6.79 (d, *J*=12.7 Hz, 0.37H), 6.53 (d, *J*=8.4 Hz, 1.14H), 6.49 (d, *J*=12.7 Hz, 0.4H), 6.30 (d, *J*=8.4 Hz, 0.67H), 5.30–5.23 (m, 1H), 4.17 (t, *J*=7.2 Hz, 1H), 3.93 (t, *J*=7.1 Hz, 1H), 3.71 (d, *J*=120.2 Hz, 6H), 3.41 (t, *J*=6.1 Hz, 1H), 3.33 (t, *J*=6.3 Hz, 1H), 3.10–2.96 (m, 1H), 2.26 (dd, *J*=11.3, 4.6 Hz, 1H), 2.15–2.05 (m, 1H), 1.99–1.84 (m, 4H), 1.78 (t, *J*=10.1 Hz, 3H), 1.60–1.33 (m, 13H), 1.33–1.24 (m, 5H), 1.12–0.99 (m, 6H), 0.84 (dd, *J*=6.5, 1.4 Hz, 4H), 0.79 (dd, *J*=6.6, 1.7 Hz, 7H), 0.60 (d, *J*=3.5 Hz, 3H). ^13^C NMR (126 MHz, CDCl_3_) δ 188.95, 171.37, 161.30, 160.26, 157.60, 141.18, 141.14, 141.01, 136.82, 135.89, 130.20, 129.37, 127.56, 126.80, 122.98, 122.92, 122.18, 121.81, 121.65, 116.28, 114.69, 109.70, 104.00, 103.57, 79.29, 79.22, 77.41, 77.36, 77.16, 76.91, 71.95, 67.49, 67.36, 67.30, 64.52, 64.02, 56.93, 56.30, 56.04, 55.54, 53.56, 50.36, 50.11, 46.80, 42.47, 39.93, 39.66, 39.31, 37.41, 37.04, 36.64, 36.33, 35.92, 33.95, 32.09, 32.07, 32.04, 31.80, 31.58, 30.33, 30.22, 29.84, 29.80, 29.76, 29.71, 29.65, 29.58, 29.50, 29.46, 29.41, 29.37, 29.30, 29.09, 28.92, 28.60, 28.37, 28.15, 27.37, 27.01, 26.77, 26.66, 25.88, 25.66, 24.43, 23.96, 22.96, 22.83, 22.70, 21.21, 21.14, 19.52, 19.30, 19.01, 18.86, 14.26, 12.11, 12.00.

### Synthesis of BODIPY (12)

300 mg of 4 hydroxy benzaldehyde (**11**) (2.45 mmol) and 500 μL of 2.4 dimethyl pyrrol (**10**) (4.85 mmol) were dissolved in 70 mL of dichloromethane under argon atmosphere. 60 μL of TFA (0.8 mmol) were added and reacted overnight. After this period, 600 mg of DDQ (2.64 mmol) were dissolved in 30 mL of dichloromethane and added to the reaction mixture and the reaction was prolonged for additional 4 hours. 15 mL of triethylamine (179 mmol) were added for more 30 minutes and then 15 mL of BF_3_.Et_2_O (121 mmol) were mixed. The reaction was stirred overnight (all steps were performed at room temperature). After the reaction was completed, it was filtered over celite pad to remove DDQ and washed with NaHCO_3_ 10 %, water then brine (one time each). The solvent was evaporated under reduced pressure forming a dark purple solid. The chromatographic column was made using dichloromethane as eluent. The product visualization was performed using UV light. The product was recovered as an orange solid (15 % yield) and showed ^1^H and ^13^C NMR spectra (Figure S9) in accordance with previous report.^[33]^

^1^H NMR (400 MHz, CDCl_3_) δ 7.13–7.09 (m, 2H), 6.97–6.93 (m, 2H), 5.97 (s, 2H), 5.65 (s, 1H), 2.54 (s, 6H), 1.44 (s, 6H). ^13^C NMR (101 MHz, CDCl_3_) δ 156.62, 155.43, 143.33, 141.98, 132.00, 129.51, 127.20, 121.26, 116.27, 14.70.

### BODIPY‐Cholesterol Conjugate (13)

K_2_CO_3_ (53 mg; 0.38 mmol) was mixed in 2 mL of dried DMF and heated to 100 °C under agitation for 1 hour. In a proper microwave flask, 10 mg of BODIPY (0.029 mmol), 16.5 mg of Cholesterol bromide (0.031 mmol) and the mixture DMF/K_2_CO_3_ were added. The flask was placed on the microwave platform and heated at 70 °C for 1 hour. After that, the solvent was removed, the reaction was purified by chromatographic column using dichloromethane as eluent. The band visualization was performed using solution of H_2_SO_4_ 10 % in methanol and UV light. Monoisotopic mass: calculated [M+H]^+^ 781.56579, found 781.56464, as demonstrated by ^1^H and ^13^C NMR (Figure S10).

^1^H NMR (400 MHz, CDCl_3_) δ 7.76–7.66 (m, 1H), 7.59–7.47 (m, 1H), 7.15 (d, *J*=8.7 Hz, 2H), 6.99 (d, *J*=8.7 Hz, 2H), 5.97 (s, 2H), 5.35 (d, *J*=5.3 Hz, 1H), 4.26 (dt, *J*=12.0, 6.4 Hz, 2H), 4.09 (d, *J*=6.7 Hz, 1H), 4.04 (t, *J*=6.4 Hz, 2H), 3.56 (t, *J*=6.4 Hz, 2H), 3.16 (td, *J*=11.2, 5.6 Hz, 1H), 2.55 (s, 6H), 2.38 (ddd, *J*=13.0, 4.5, 1.9 Hz, 1H), 2.25–2.19 (m, 1H), 2.02 (dd, *J*=13.1, 4.1 Hz, 2H), 1.90 (dd, *J*=14.8, 6.1 Hz, 4H), 1.43 (s, 6H), 1.25 (s, 7H), 1.18–1.04 (m, 10H), 1.01 (s, 3H), 0.99–0.88 (m, 12H), 0.86 (dd, *J*=6.6, 1.7 Hz, 8H), 0.68 (s, 3H). ^13^C NMR (126 MHz, CDCl_3_) δ 167.77, 159.65, 155.22, 143.19, 141.98, 141.05, 131.88, 130.91, 129.83, 129.16, 128.83, 126.88, 121.56, 121.08, 115.07, 79.09, 71.81, 68.17, 67.90, 67.55, 65.58, 63.13, 56.81, 56.18, 50.24, 42.34, 39.81, 39.53, 39.23, 38.74, 37.29, 36.94, 36.20, 35.80, 32.82, 31.97, 31.94, 31.92, 30.58, 30.37, 29.71, 29.67, 29.63, 29.53, 29.51, 29.45, 29.41, 29.37, 29.33, 29.24, 29.09, 28.94, 28.52, 28.24, 28.03, 27.73, 27.23, 26.77, 26.23, 25.75, 24.76, 24.30, 23.83, 23.76, 22.99, 22.83, 22.70, 22.57, 21.09, 19.40, 19.19, 19.17, 18.73, 14.59, 14.13, 14.06, 13.74, 11.87, 10.97.

### Biology

**Cells and reagents for cell culture**: HEK293‐*ABCG2* (stably transfected) and HEK293 (wild‐type) cells were cultivated in Dulbecco's modified Eagle's medium High Glucose (Gibco, NY, USA) supplemented with 10 % of bovine fetal serum (FBS) (Gibco, NY, USA), Penicillin/Streptomycin 1 % (Gibco, NY, USA) and amphotericin B 0.25 μg/mL (Gibco, NY, USA). Cells were maintained at 37 °C at 5 % of CO_2_ until 80–90 % of confluence and detached for experiments using Trypsin‐EDTA 0.05 % (Gibco, NY, USA). Substrate hoechst 33342 was purchased from Invitrogen (Oregon, USA), mitoxantrone from Sigma‐Aldrich (Massachusetts, USA) and pheophorbide *a* was synthetized as described. ABCG2 inhibitor Ko143 was purchased from Abcam (Cambridge, UK).

**Cholesterol influence on ABCG2‐mediated transport of substrates**: HEK293‐*ABCG2* cells were seeded on 24 well plates (2.5×10^5^) and incubated for 24 hours for adhesion. Then, cells were treated as follow: mitoxantrone (0.15–20 μM), hoechst 33342 (0.09–12 μM) or pheophorbide *a* (0.15–20 μM) in absence and presence of cholesterol (50 μM). Experimental control conditions were performed using mitoxantrone (7.5 μM) or hoechst 33342 (3 μM) or pheophorbide *a* (7.5 μM) and Ko143 (1 μM). The assay was analyzed by flow cytometry (FACS Celesta) using V780 – 60 filter for mitoxantrone, UV 450–50 filter for hoechst 33342 and B 695–40 for pheophorbide *a*.

**Intracellular accumulation of BODIPY and BODIPY‐Cholesterol conjugate**: To evaluate the intracellular accumulation of compounds, the fluorescence of BODIPY was quantified by flow cytometry in HEK293 cells. Cells were seeded on 24 well plates (2.5×10^5^) and incubated for 24 hours for adhesion. Then, cells were treated with increasing concentrations of BODIPY (0.25–4 μM) or BODIPY – cholesterol (0.25–40 μM) for 30 minutes at 37 °C. After incubation, the supernatant was removed and attached cells were washed with PBS prior to detachment with trypsin. Cells were resuspended on cold PBS and analyzed by flow cytometry using filter B 530–30 (FACS Celesta).

**Effect of chalcone and chalcone–cholesterol conjugate on ABCG2‐mediated transport**: HEK293‐*ABCG2* cells were seeded on 24 well plates (2.5×10^5^) and incubated for 24 hours for adhesion. Cells were tested in four conditions as follow: chalcone (0.25–20 μM) and hoechst 33342 (3 μM); chalcone–cholesterol conjugate (0.25–20 μM) and hoechst 33342 (3 μM); chalcone (0.25–20 μM), cholesterol (50 μM) and hoechst 33342 (3 μM); chalcone (0.25–20 μM), cholesterol (0.25–20 μM) and hoechst 33342 (3 μM). Cells were treated for 30 minutes at 37 °C. Then, the supernatant was removed, cells were detached and resuspended in cold PBS. The analysis was performed by flow cytometry (FACS Celesta) using UV 450–50 filter.

**BODIPY‐cholesterol efflux mediated by ABCG2**: HEK293‐*ABCG2* cells were seeded on 24 well plates (1.5×10^5^) and incubated for 72 hours util 90 % of confluence. Then, cells were treated as follow for 30 minutes: hoechst 33342 (3 μM) or hoechst 33342 (3 μM)/Ko143 (1 μM); BODIPY‐cholesterol (8 μM) or BODIPY‐cholesterol (8 μM)/Ko 143 (1 μM). After 30 minutes of treatment, cells were washed with PBS followed by medium replacement without substrate or medium with Ko143 (1 μM). Cells were kept under the cited conditions in different periods of time (30 minutes, 1, 2, 4 and 6 hours). Then, the medium was removed, cells were detached with trypsin, resuspended in PBS, and analyzed by flow cytometry (UV 450–50 for hoechst 33342 and B 530–30 for BODIPY‐cholesterol). To evaluate the long‐term efflux, cells HEK293‐*ABCG2* cells were seeded on 24 well plates (1.5×10^5^). After 24 hours, cells were treated with BODIPY‐cholesterol (8 μM) or BODIPY‐cholesterol (8 μM)/Ko143 (1 μM) for 30 minutes. After, the treatment was removed and the cells were washed with PBS followed by medium replacement with Ko143 (1 μM) or medium free of tested compounds. Cells were kept under the cited conditions in different periods of time (48 and 72 hours). The analysis was performed as described above.

**Co‐treatment of cells with free cholesterol and BODIPY‐cholesterol**: HEK*293‐ABCG2* cells were seeded at a density of 2×10^5^ per well in a 24‐well culture plate and incubated at 37 °C in an atmosphere containing 5 % of CO_2_. After 48 h, cells were exposed to increased concentrations of cholesterol (0, 8, 48, 80 μM of cholesterol) and a fixed concentration of 8 μM to the BODIPY‐cholesterol conjugate. Cells were incubated for 30 minutes and then analyzed by flow cytometry using B 530–30 filter.

**Confocal microscopy**: BODIPY‐cholesterol intracellular localization was assessed in HEK293‐*ABCG2* cell line in confocal microscopy. Cells were seeded at density 1.5×10^5^ cells/well on a coverslip in a 24 well‐plate. Cells were incubated for 48 h at 37 °C containing 5 % of CO_2_. After cell adhesion to the coverslips, they were exposed to different compounds (hoechst 33342 3 μM; pheophorbide *a* 20 μM; BODIPY‐cholesterol 20 μM; Ko143 1 μM) and incubated for 30 min. After incubation, cells were washed with PBS. The coverslips were removed from the plate and placed in a slide with mounting media of glycerol 100 %. The slides were seal with nail polish. The slides were analyzed in the confocal microscopy Nikon A1R MP+ (NIKON, Tokyo, Japan) with a pinhole size of 43.42 μm. Images were taken using the same capture characteristics, with an objective of 60× (numerical aperture of 1.27) in water immersion, at a scan speed of 0.25 fps and magnification of 0.5×. Hoechst 33342 was excited by a laser of 405 nm (laser power:10 %, PMT gain: 120 V, PMT offset: −10 V) and the fluorescence detection by the bandpass filter of 452/45. BODIPY‐cholesterol was excited by a laser of 488 nm (laser power:10 %, PMT gain: 110 V, PMT offset: −10 V) and the fluorescence detection by the bandpass filter of 525/50. Pheophorbide *a* was excited by a laser of 405 nm (laser power:10 %, PMT gain: 115 V, PMT offset: −10 V) and the fluorescence detection by the bandpass filter of 593/46. The images were prepared in ImageJ 1.53 t software version 1.8_0322 (National Institute of Health, USA).^[38]^

## Conflict of Interests

The authors declare no conflict of interest.

1

## Supporting information

As a service to our authors and readers, this journal provides supporting information supplied by the authors. Such materials are peer reviewed and may be re‐organized for online delivery, but are not copy‐edited or typeset. Technical support issues arising from supporting information (other than missing files) should be addressed to the authors.

Supporting Information

## Data Availability

The data that support the findings of this study are available in the supplementary material of this article and through the corresponding authors.
