# Supplementary material for: Effects of Cholesterol on the Breast Cancer Resistance Protein: Studies through the Synthesis and Biological Evaluation of Chemical Tools
Source: ChemMedChem. 2025 Jan 31;20(8):e202400712. doi: 10.1002/cmdc.202400712 (PMC12005470; doi:10.1002/cmdc.202400712)
Supplement: Supplementary file 1 — Supporting Information [file CMDC-20-e202400712-s001.pdf]

ChemMedChem

Supporting Information

Effects of Cholesterol on the Breast Cancer Resistance Protein: Studies through the Synthesis and Biological Evaluation of Chemical Tools

Ingrid Fatima Zattoni, Bruna Estelita Rugisnk, Isadora da Silva Zanzarini, Alan Guilherme Gonçalves, Vivian Rotuno Moure, Glaucio Valdameri,* and Ahcène Boumendjel*

Supporting Information

Effects of Cholesterol on the Breast Cancer Resistance Protein: Studies through the Synthesis and Biological Evaluation of Chemical Tools

Fig. S1. Confocal microscopy panel with all tested channels and substrates.

Fig. S2. Fluorescence shift according to concentrations used for BODIPY and BODIPY–cholesterol using blue laser and filter 530-30. The data represents one experiment.

Fig. S3. Effect of exposure to free-cholesterol on the cellular accumulation of the BODIPY-cholesterol conjugate.

Fig. S4. Pheophytin chemical characterization (NMR^{1}H and Mass spectroscopy)

Fig. S5. Pheophorbide *a* chemical characterization (NMR^{1}H and Mass spectroscopy)

Fig. S6. Chalcone chemical characterization (NMR^{1}H, NMR^{13}C and Mass spectroscopy)

Fig. S7. Cholesterol *O*-alkylated chemical characterization (NMR^{1}H and NMR^{13}C)

Fig. S8. Chalcone-cholesterol conjugate chemical characterization (NMR^{1}H, NMR^{13}C and Mass spectroscopy)

Fig. S9. BODIPY chemical characterization (NMR^{1}H, NMR^{13}C and Mass spectroscopy)

Fig. S10. BODIPY- Cholesterol heterodimer chemical characterization (NMR^{1}H, NMR^{13}C and Mass spectroscopy)

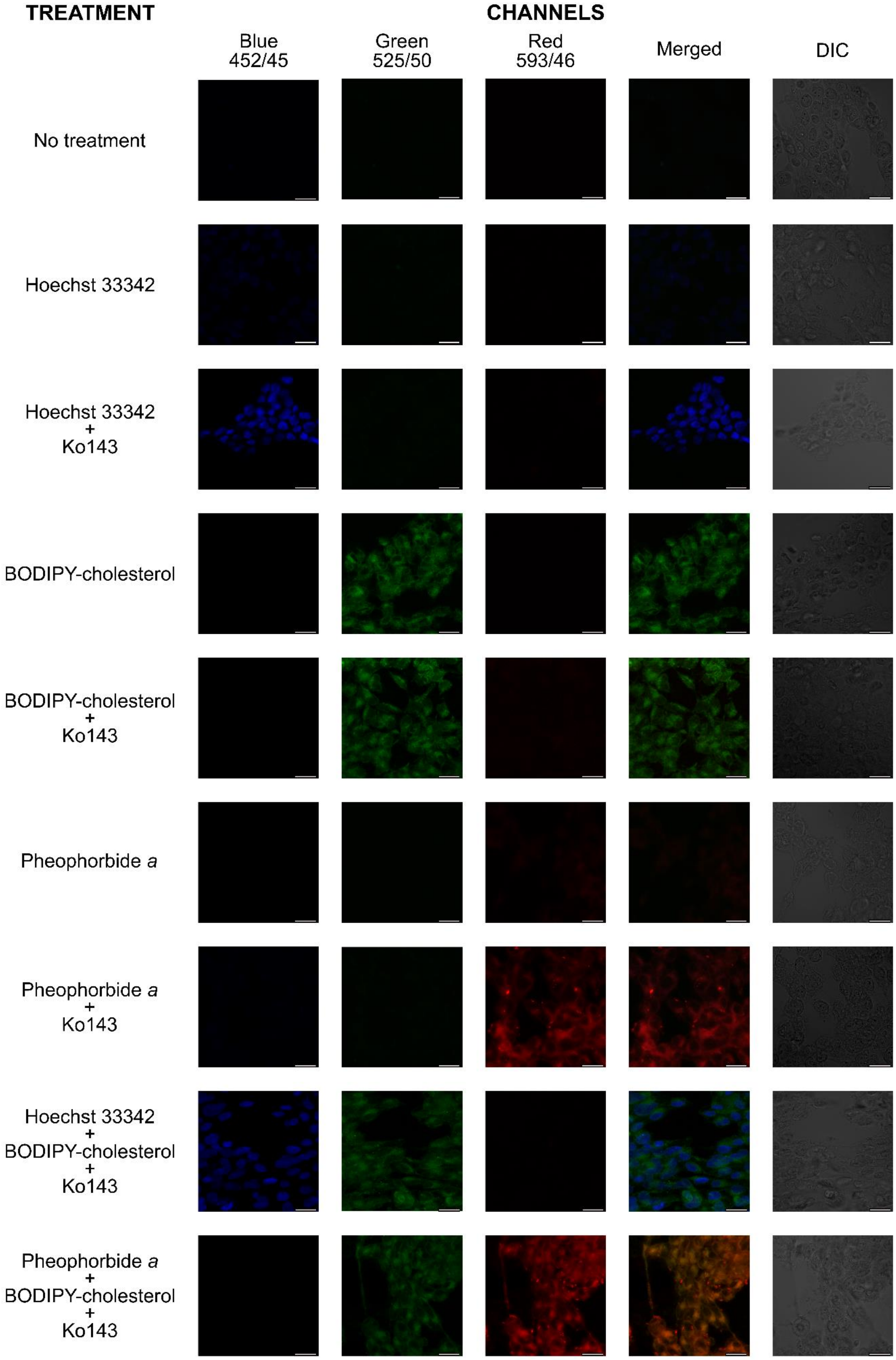

Fig. S1. Confocal microscopy panel with all tested channels and substrates.

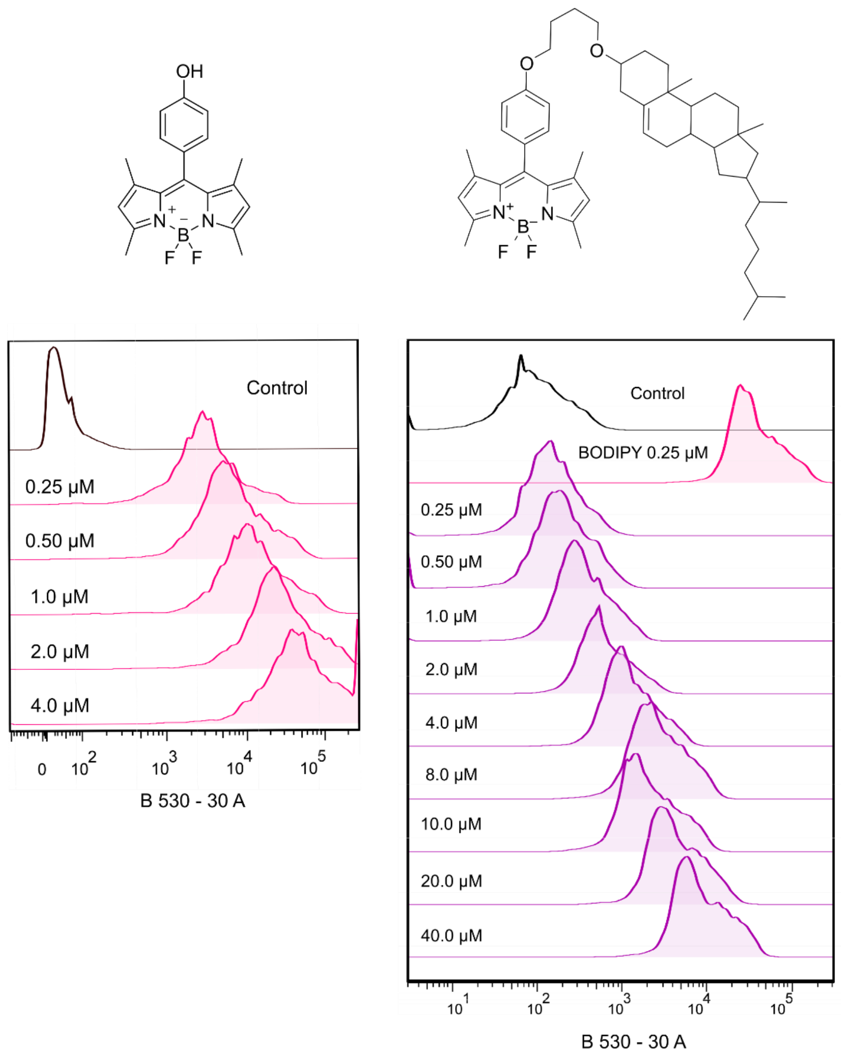

Fig. S2. Fluorescence shift according to concentrations used for BODIPY and BODIPY–cholesterol using blue laser and filter 530-30. The data represents one experiment.

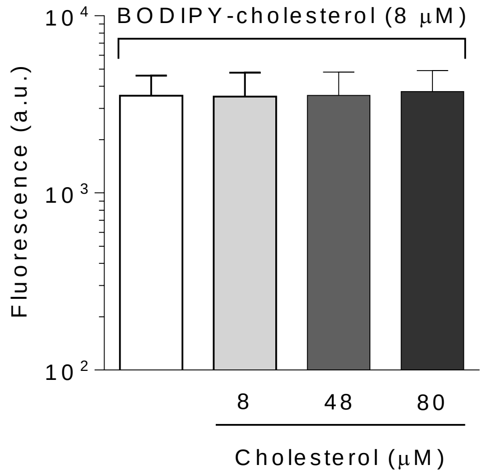

Fig. S3. Effect of exposure to free-cholesterol on the cellular accumulation of the heterodimer BODIPY-cholesterol. Cells were exposed to increased concentrations of free-cholesterol (8 µM, 48 µM and 80 µM), and the heterodimer BODIPY-cholesterol at 8 µM. Intracellular accumulation was quantified by low cytometry. Data represent the mean ± SD of two independent experiments.

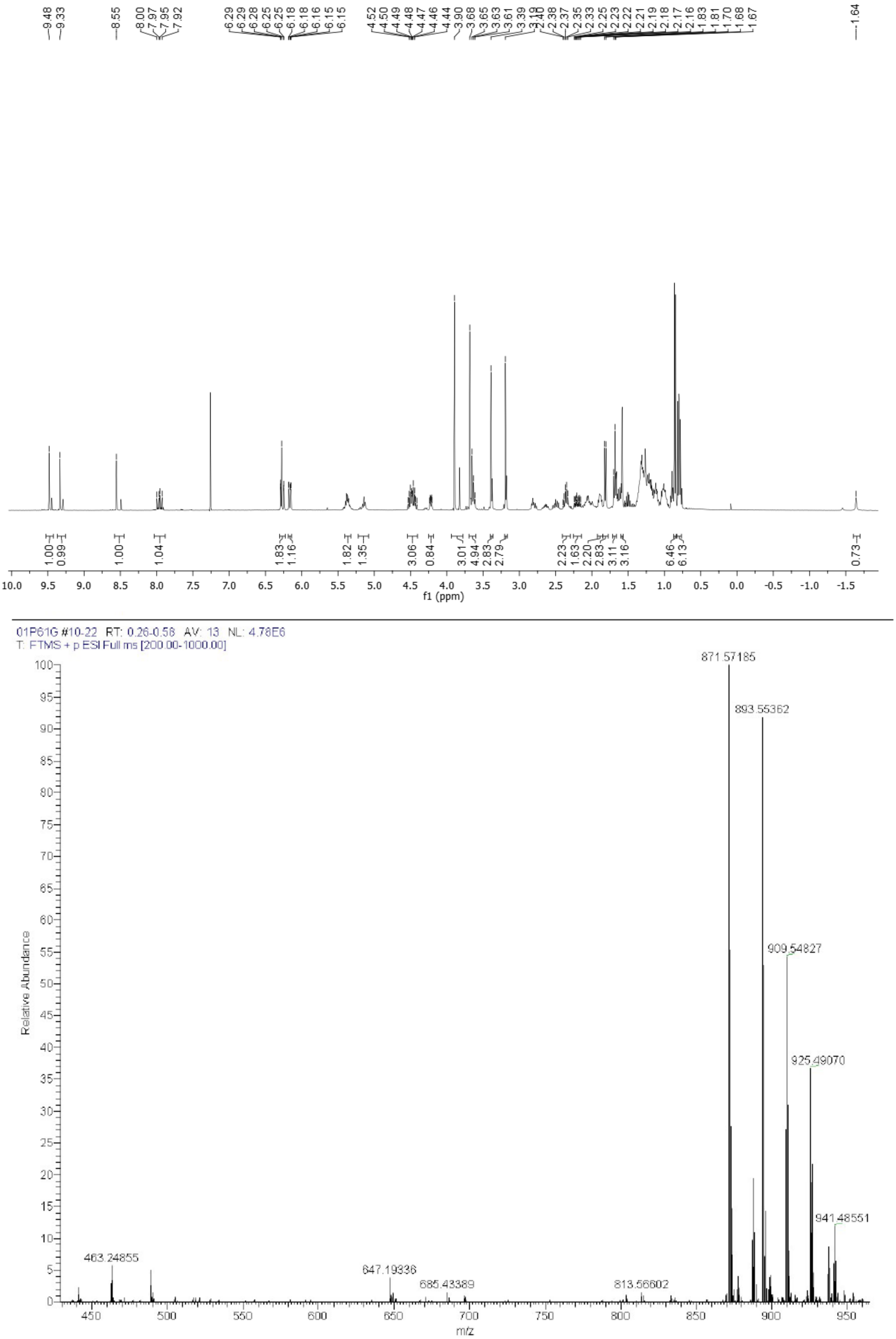

Fig. S4. Pheophytin chemical characterization (NMR^{1}H and Mass spectroscopy)

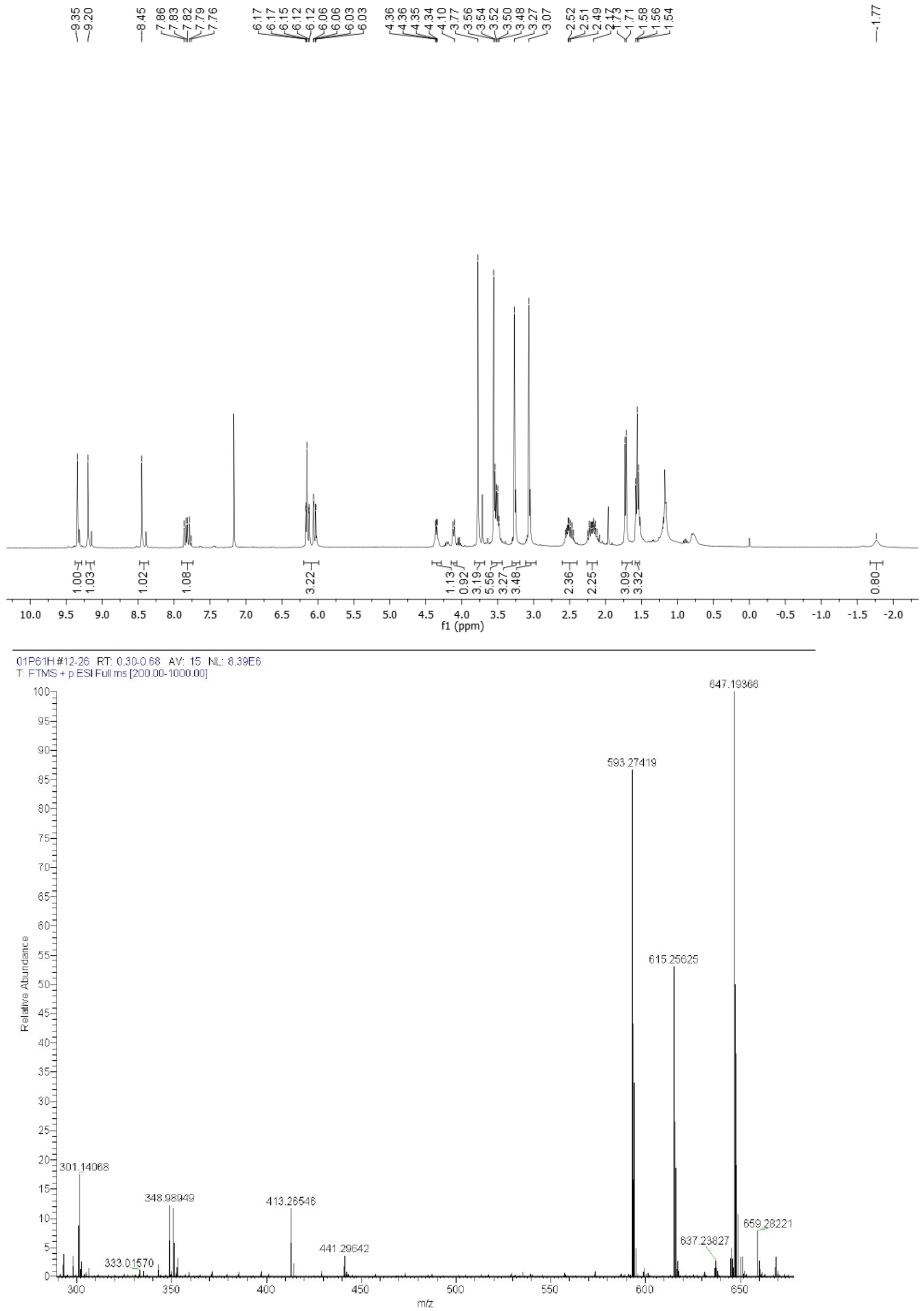

Fig. S5. Pheophorbide *a* chemical characterization (NMR^{1}H and Mass spectroscopy)

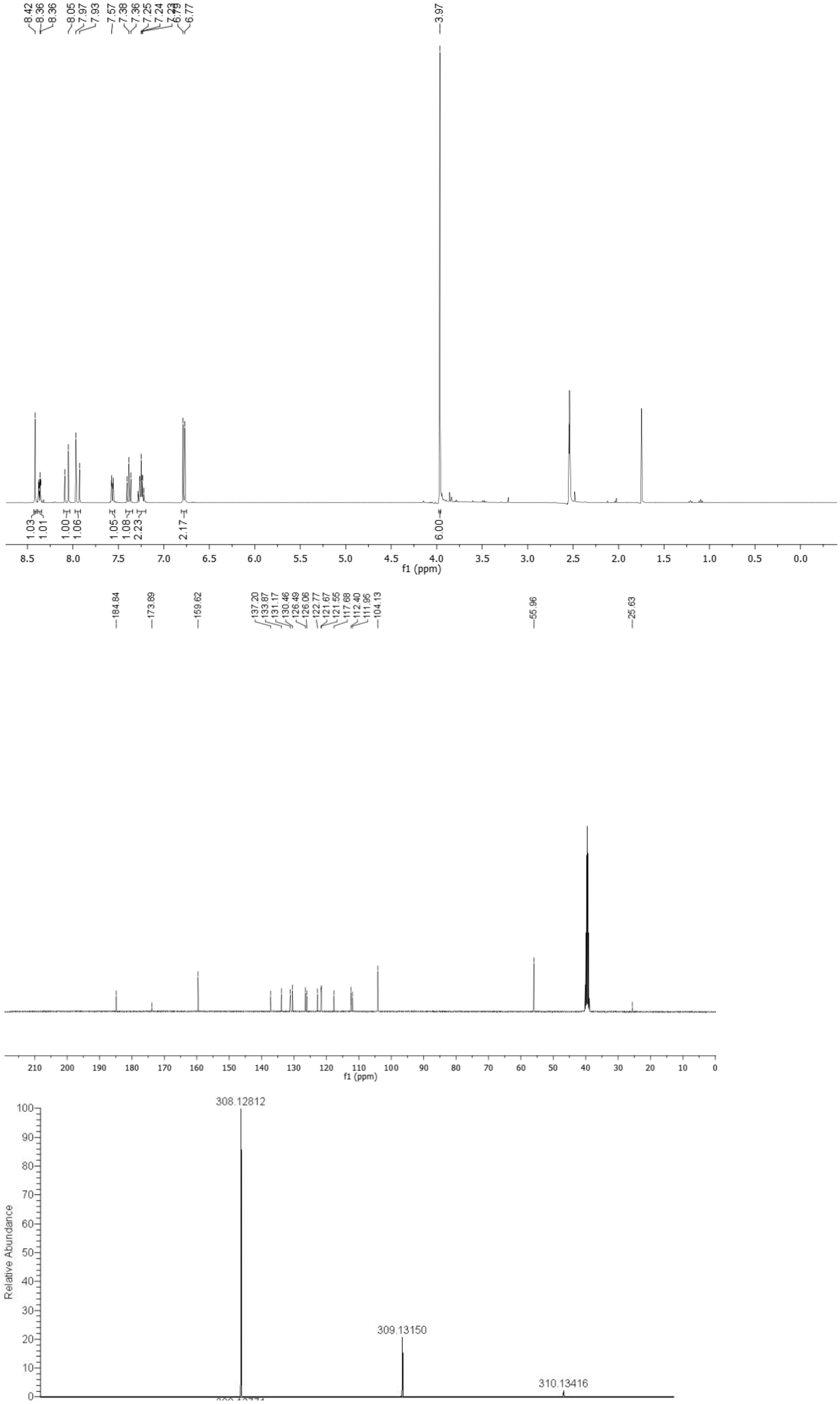

Fig. S6. Chalcone chemical characterization (NMR^{1}H, NMR^{13}C and Mass spectroscopy)

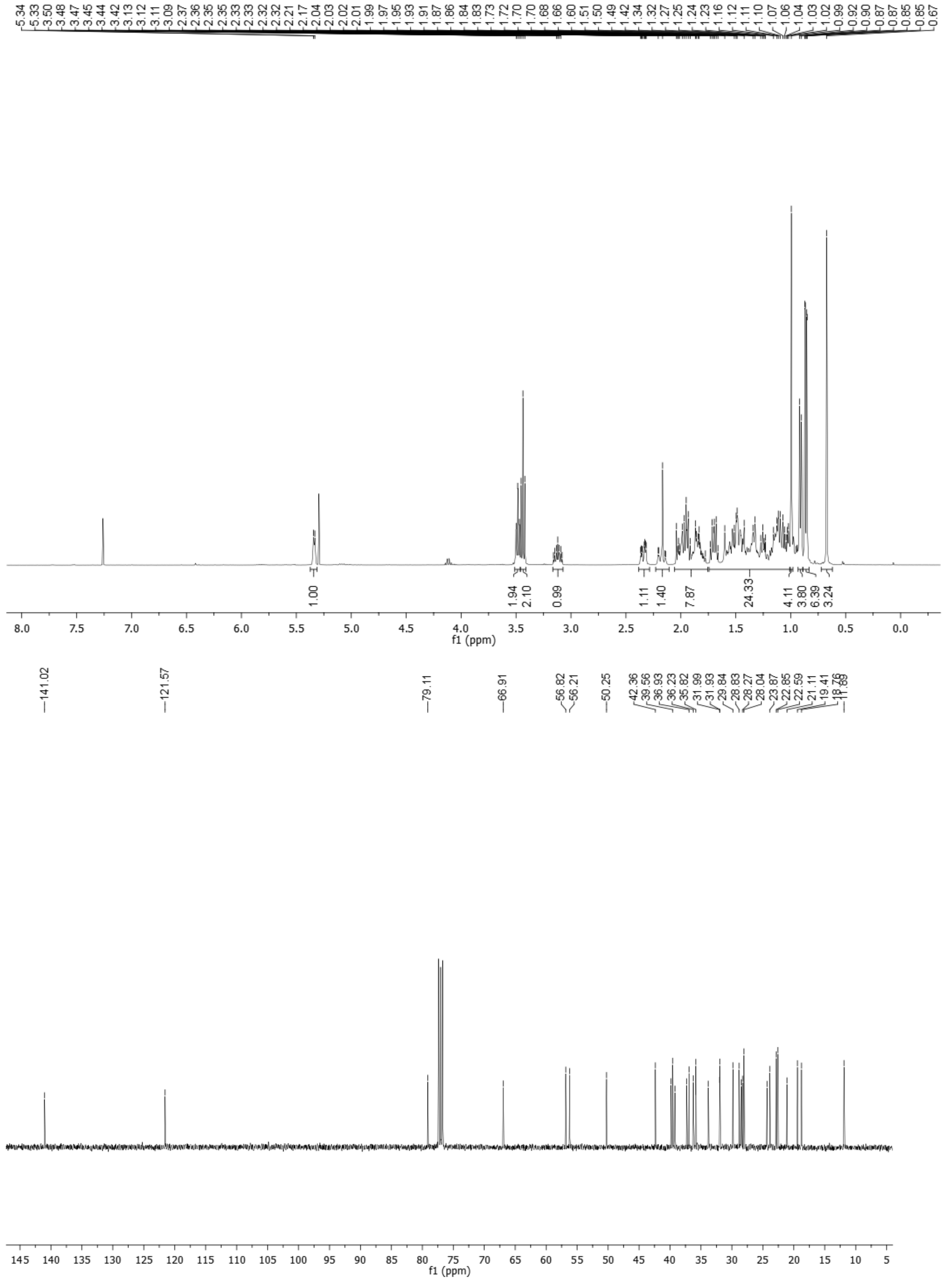

Fig. S7. Cholesterol *O*-alkylated chemical characterization (NMR^{1}H and NMR^{13}C)

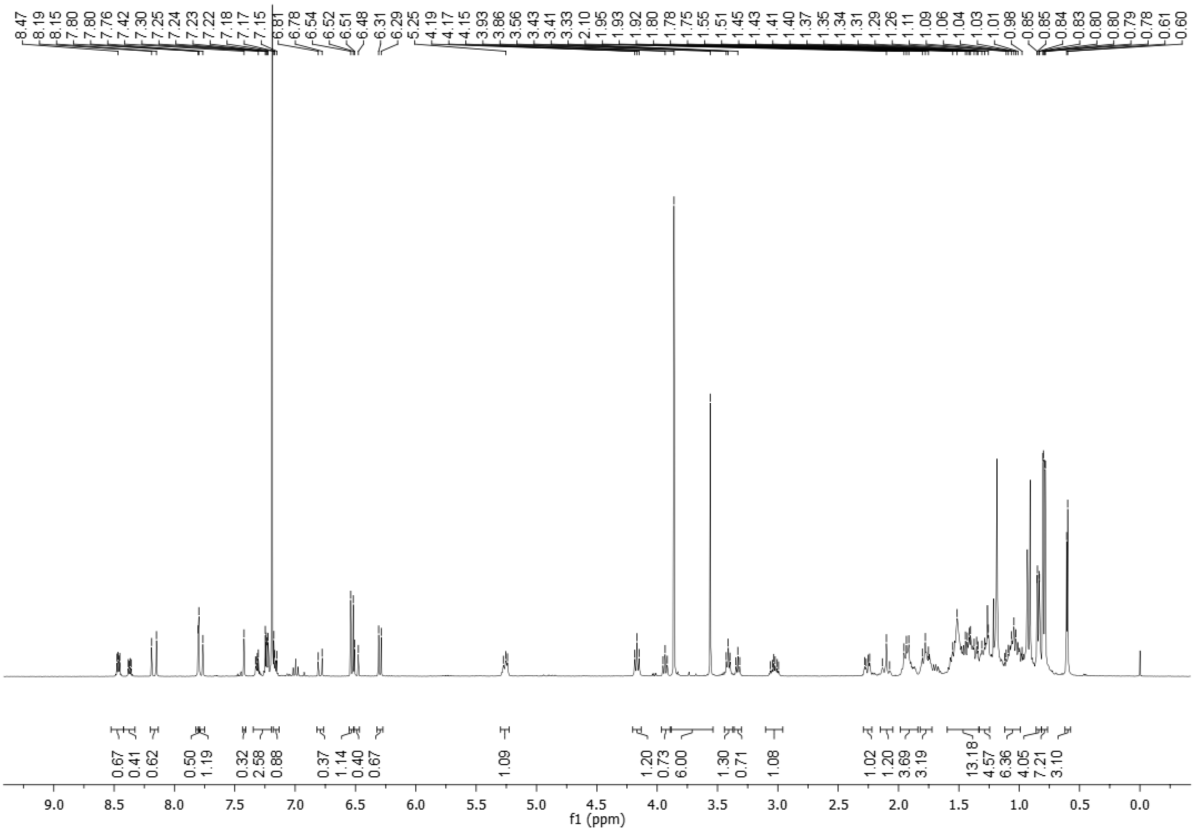

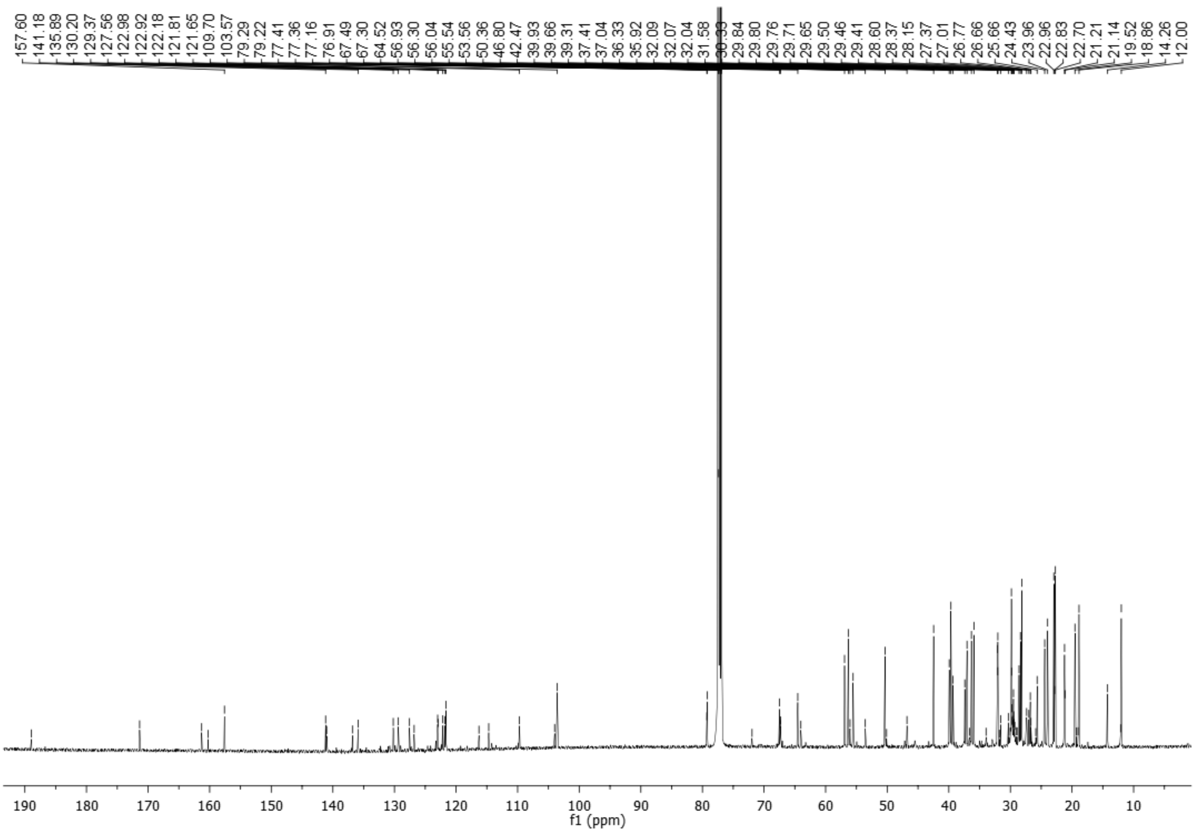

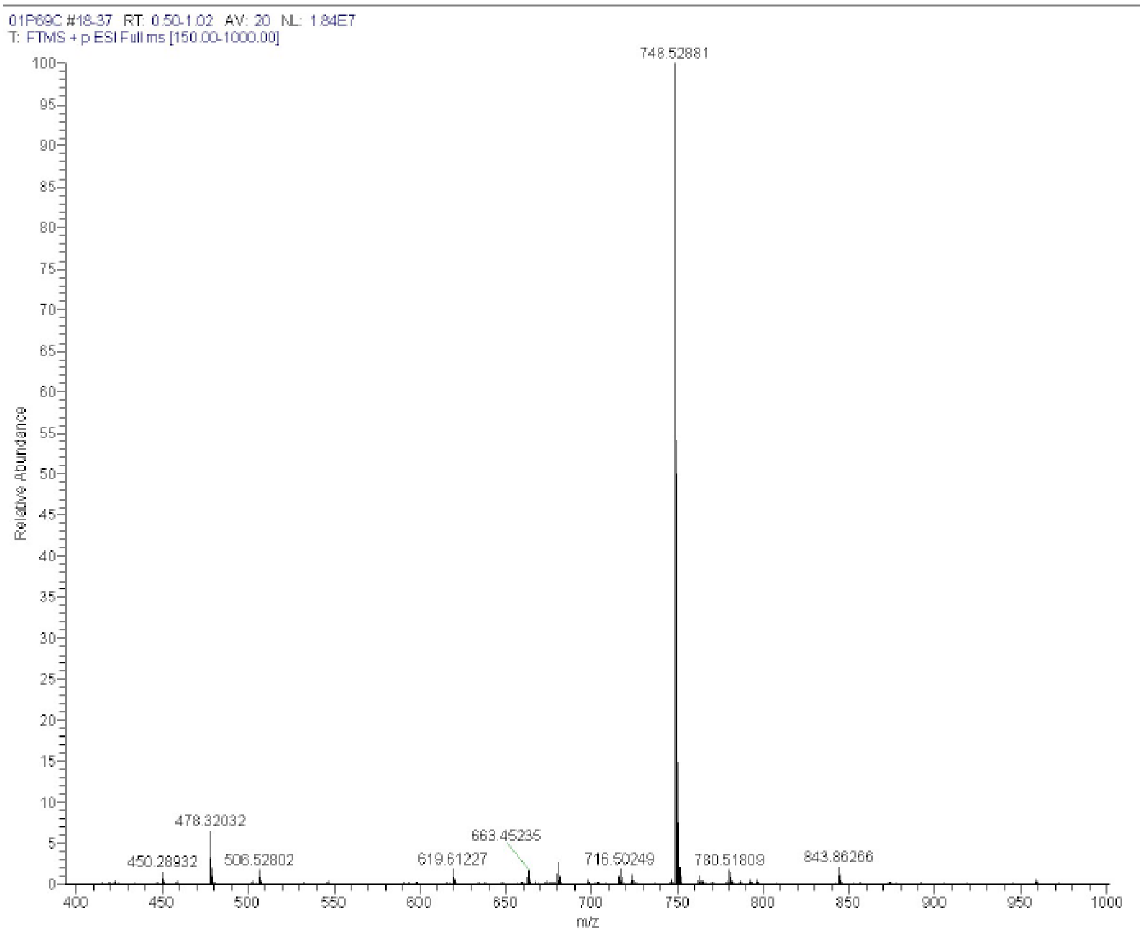

Fig. S8. Chalcone-cholesterol conjugate chemical characterization (NMR^{1}H, NMR^{13}C and Mass spectroscopy)

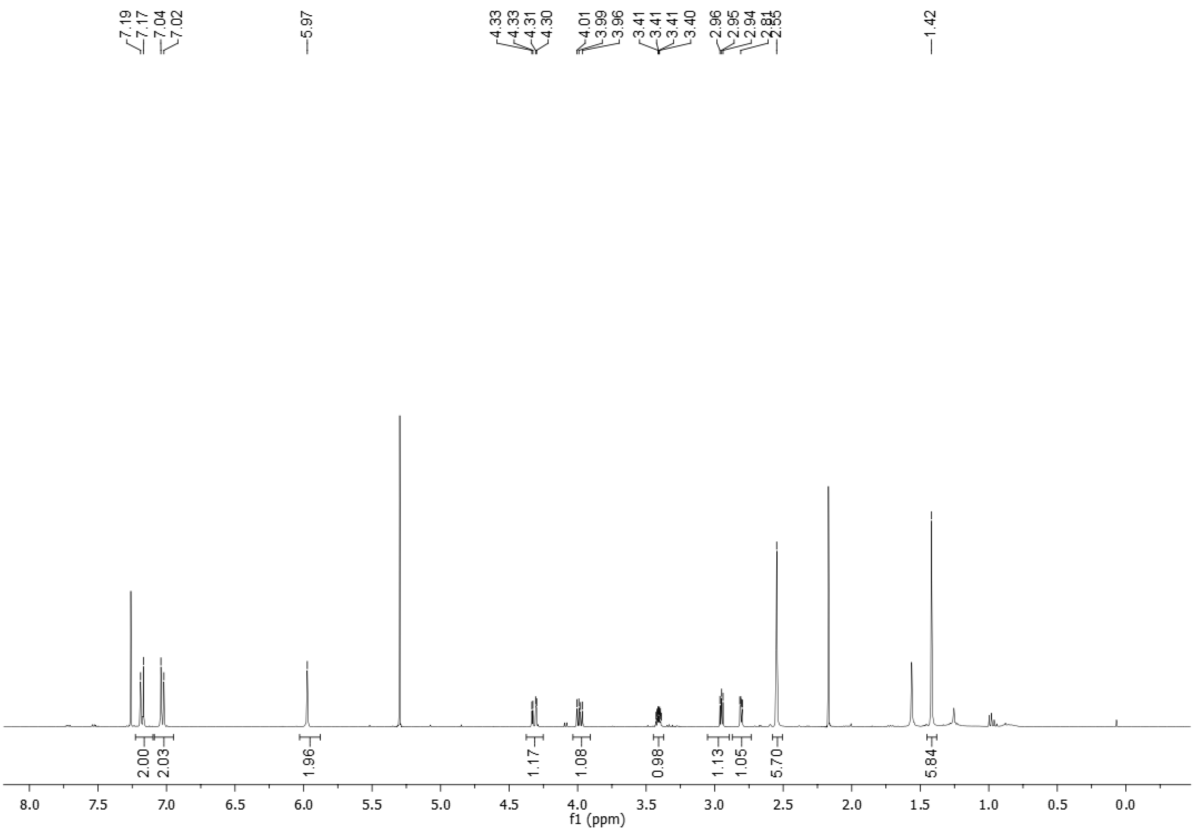
7.19
7.17
7.04
7.02
5.97
4.33
4.33
4.31
4.30
4.01
3.99
3.96
3.41
3.41
3.41
3.40
2.96
2.95
2.94
2.81
2.55
1.42
2.00
2.03
1.96
1.17
1.08
0.98
1.13
1.05
5.70
5.84
8.0
7.5
7.0
6.5
6.0
5.5
5.0
4.5
4.0
3.5
3.0
2.5
2.0
1.5
1.0
0.5
0.0
f1 (ppm)

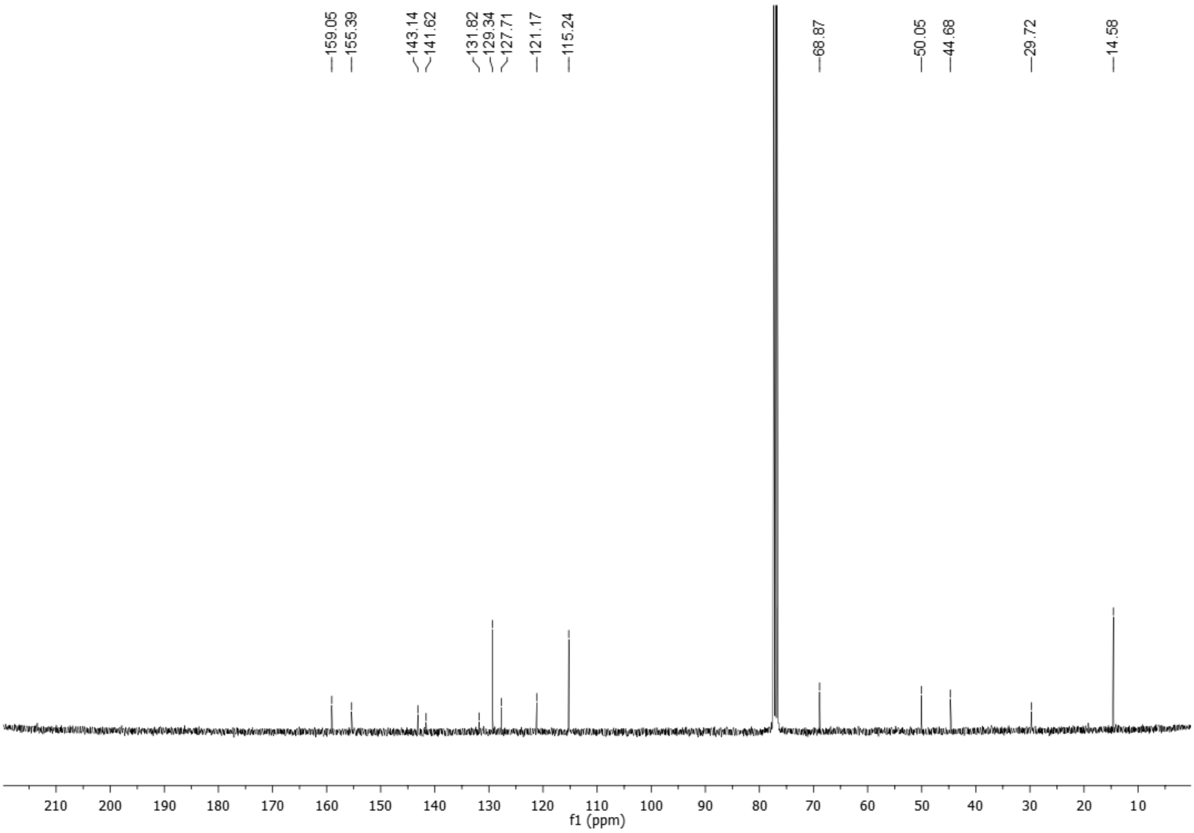
159.05
155.39
143.14
141.62
131.82
129.34
127.71
121.17
115.24
68.87
50.05
44.68
29.72
14.58
210
200
190
180
170
160
150
140
130
120
110
100
90
80
70
60
50
40
30
20
10
f1 (ppm)

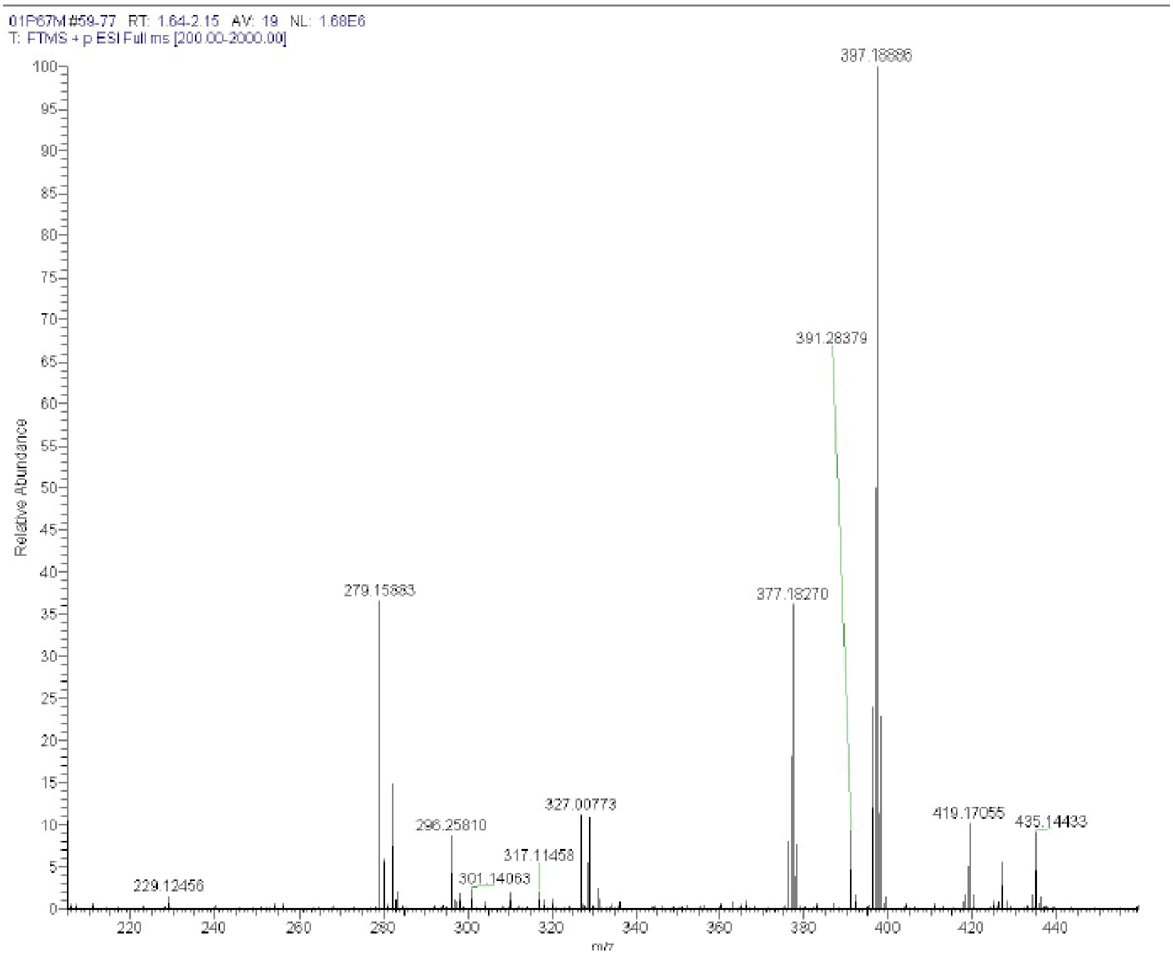

Fig. S9. BODIPY chemical characterization (NMR^{1}H, NMR^{13}C and Mass spectroscopy)

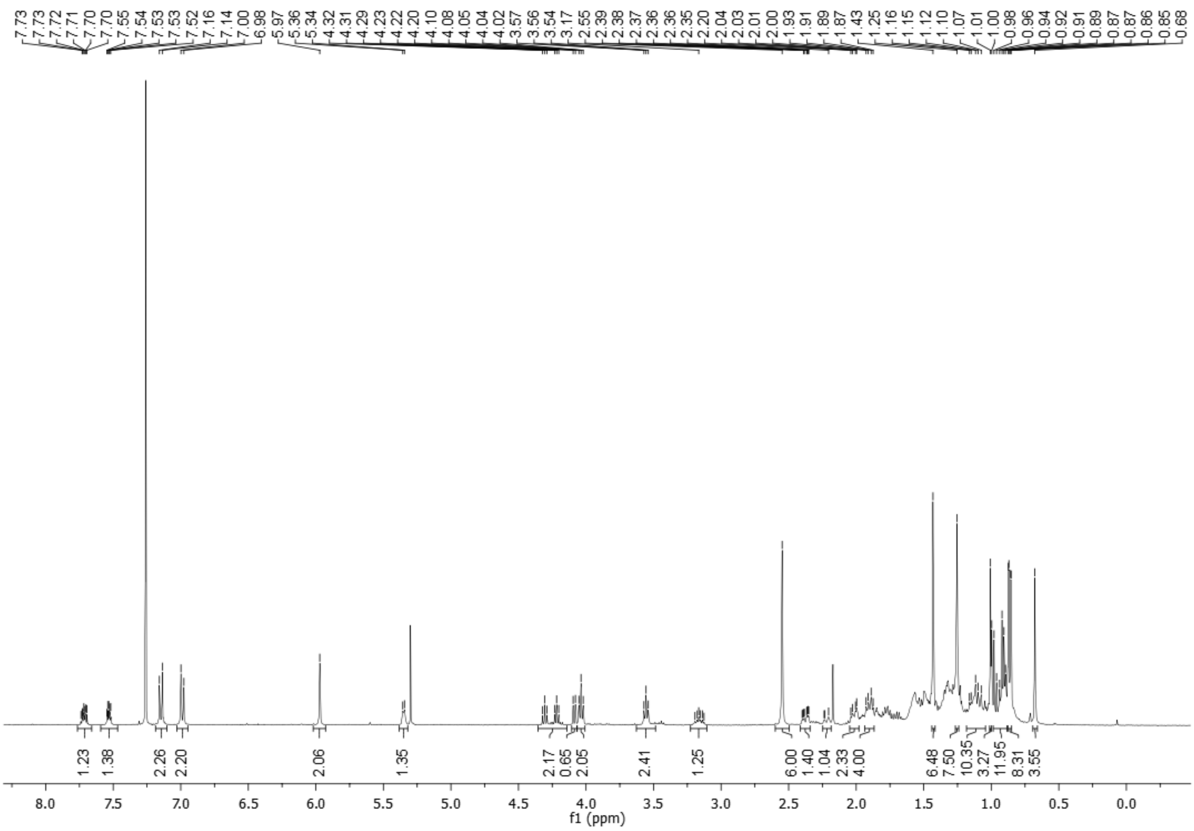
7.73 7.73 7.72 7.71 7.70 7.70 7.55 7.54 7.53 7.53 7.52 7.16 7.14 7.00 6.98 5.97 5.36 5.34 4.32 4.31 4.29 4.23 4.22 4.20 4.10 4.08 4.05 4.04 4.02 3.57 3.56 3.54 3.17 2.55 2.39 2.38 2.37 2.36 2.35 2.20 2.04 2.03 2.01 2.00 1.93 1.91 1.89 1.87 1.43 1.25 1.16 1.15 1.12 1.10 1.07 1.01 1.00 0.98 0.96 0.94 0.92 0.91 0.89 0.87 0.86 0.85 0.68
1.23 1.38 2.26 2.20 2.06 1.35 2.17 0.65 2.05 2.41 1.25 6.00 1.40 1.04 2.33 4.00 6.48 7.50 10.35 3.27 11.95 8.31 3.55
8.0 7.5 7.0 6.5 6.0 5.5 5.0 4.5 4.0 3.5 3.0 2.5 2.0 1.5 1.0 0.5 0.0
f1 (ppm)

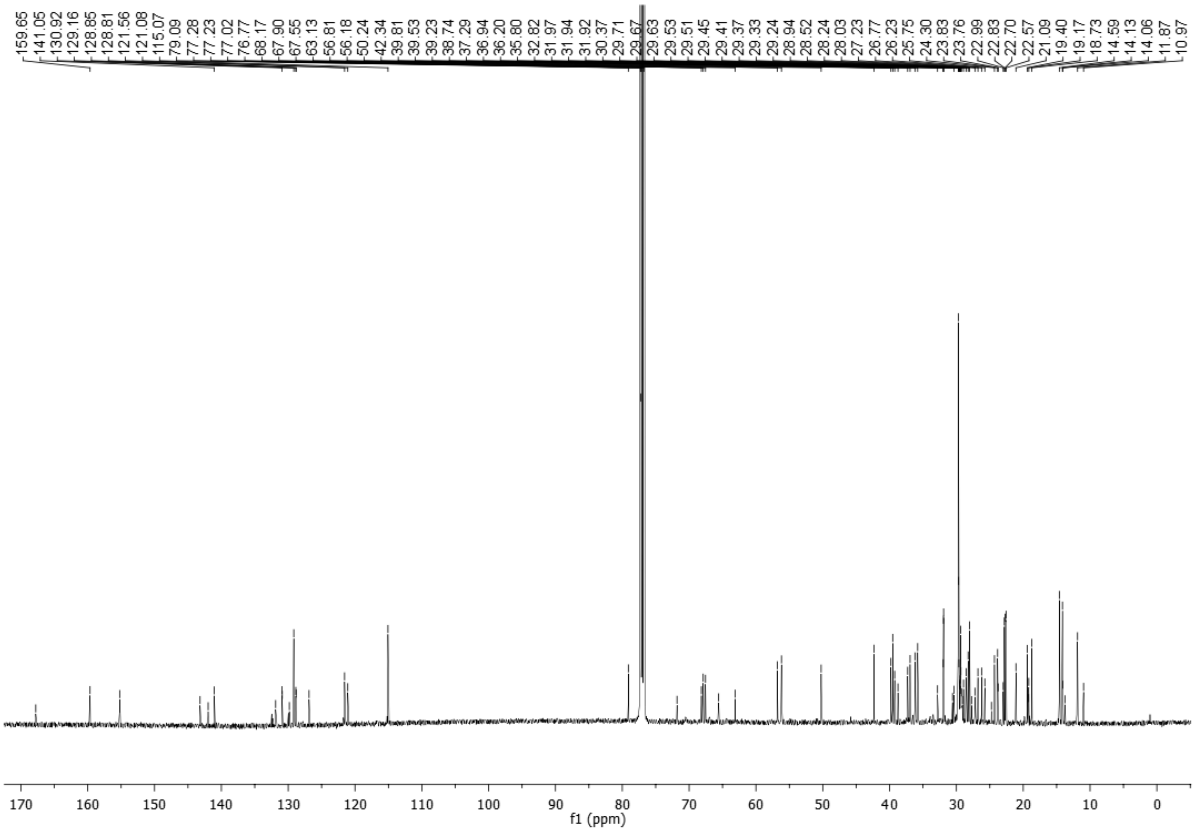
159.65 141.05 130.92 129.16 128.85 128.81 121.56 121.08 115.07 79.09 77.28 77.23 77.02 76.77 68.17 67.90 67.55 63.13 56.81 56.18 50.24 42.34 39.81 39.53 39.23 38.74 37.29 36.94 36.20 35.80 32.82 31.97 31.94 31.92 30.37 29.71 29.67 29.63 29.53 29.51 29.45 29.41 29.37 29.33 29.24 28.94 28.52 28.24 28.03 27.23 26.77 26.23 25.75 24.30 23.83 23.76 22.99 22.83 22.70 22.57 21.09 19.40 19.17 18.73 14.59 14.13 14.06 11.87 10.97
170 160 150 140 130 120 110 100 90 80 70 60 50 40 30 20 10 0
f1 (ppm)

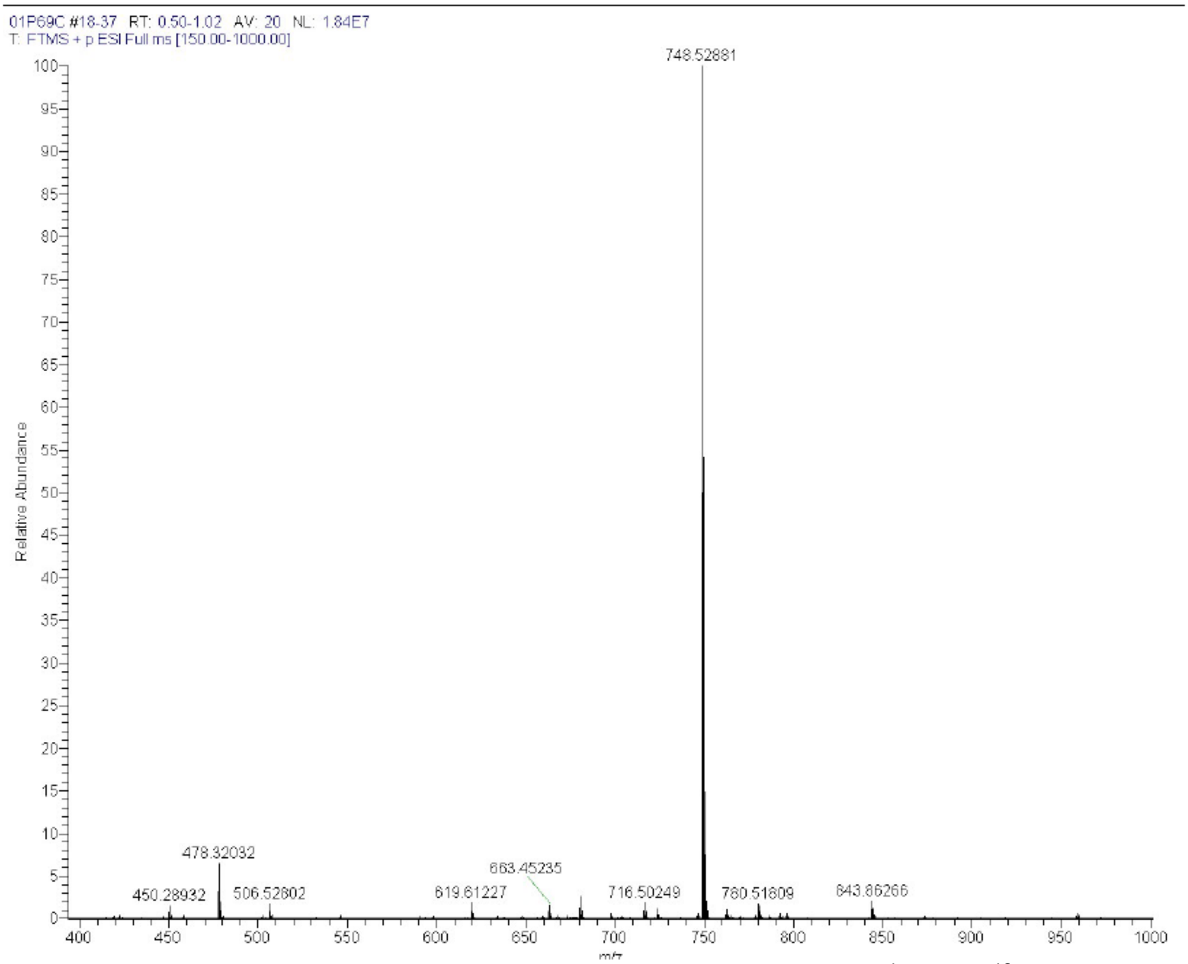

Fig. S10. BODIPY- Cholesterol heterodimer chemical characterization (NMR^{1}H, NMR^{13}C and Mass spectroscopy)
